# Chemosynthetic symbionts of marine invertebrate animals are capable of nitrogen fixation

**DOI:** 10.1038/nmicrobiol.2016.195

**Published:** 2016-10-24

**Authors:** Jillian M. Petersen, Anna Kemper, Harald Gruber-Vodicka, Ulisse Cardini, Matthijs van der Geest, Manuel Kleiner, Silvia Bulgheresi, Marc Mußmann, Craig Herbold, Brandon K.B. Seah, Chakkiath Paul Antony, Dan Liu, Alexandra Belitz, Miriam Weber

**Affiliations:** 1 Department of Microbiology and Ecosystem Science, Division of Microbial Ecology, Research Network Chemistry meets Microbiology, University of Vienna, Althanstrasse 14, Vienna, 1090 Austria; 2 Max Planck Institute for Marine Microbiology, Celsiusstrasse 1, Bremen, 28359 Germany; 3 Centre for Marine Biodiversity, Exploitation and Conservation (MARBEC), UMR 9190, IRD-IFREMER-CNRS-UM, Université de Montpellier, Montpellier Cedex 5, 34095 France; 4 Department of Coastal Systems and Utrecht University, NIOZ Royal Netherlands Institute for Sea Research, PO Box 59, 1790 AB Den Burg, Texel, The Netherlands; 5 Department of Geoscience, University of Calgary, 2500 University Drive Northwest, Alberta, T2N 1N4 Canada; 6 Archaea Biology and Ecogenomics Division, Department of Ecogenomics and Systems Biology, University of Vienna, Althanstrasse 14, Vienna, 1090 Austria; 7 HYDRA Institute for Marine Sciences, Elba Field Station, Campo nell'Elba, Livorno, 54037 Italy

**Keywords:** Microbial ecology, Symbiosis, Water microbiology, Biogeochemistry

## Abstract

Chemosynthetic symbioses are partnerships between invertebrate animals and chemosynthetic bacteria. The latter are the primary producers, providing most of the organic carbon needed for the animal host's nutrition. We sequenced genomes of the chemosynthetic symbionts from the lucinid bivalve *Loripes lucinalis* and the stilbonematid nematode *Laxus oneistus*. The symbionts of both host species encoded nitrogen fixation genes. This is remarkable as no marine chemosynthetic symbiont was previously known to be capable of nitrogen fixation. We detected nitrogenase expression by the symbionts of lucinid clams at the transcriptomic and proteomic level. Mean stable nitrogen isotope values of *Loripes lucinalis* were within the range expected for fixed atmospheric nitrogen, further suggesting active nitrogen fixation by the symbionts. The ability to fix nitrogen may be widespread among chemosynthetic symbioses in oligotrophic habitats, where nitrogen availability often limits primary productivity.

**Supplementary information:**

The online version of this article (doi:10.1038/nmicrobiol.2016.195) contains supplementary material, which is available to authorized users.

## Abstract (web summary)

The chemosynthetic symbionts of the bivalve *Loripes lucinalis* and nematode *Laxus oneistus* are found to encode nitrogen fixation genes, with evidence for active nitrogen fixation.

**Supplementary information:**

The online version of this article (doi:10.1038/nmicrobiol.2016.195) contains supplementary material, which is available to authorized users.

Symbioses between animals and chemosynthetic bacteria are widespread in Earth's oceans^1^. Animals from at least seven phyla have formed such symbioses, and even more chemosynthetic bacterial lineages have evolved symbioses with animal hosts^1^. Chemosynthetic symbionts can use a range of chemicals, such as sulfide, methane, hydrogen and carbon monoxide, to power their metabolism^2–4^. The hosts of chemosynthetic symbionts dominate some animal communities. For example, shallow-water lucinid bivalves, which host sulfur-oxidizing symbionts, often dominate the macrobenthic infaunal community in seagrass meadows, where they can reach densities greater than 3,500 individuals per square metre^5,6^. Their diversity in nature, their persistence over evolutionary timescales and their dominance in many habitats attest to the success of these symbiotic partnerships^1^.

Chemosynthetic symbionts are primarily considered ‘nutritional symbionts’, meaning their primary role is to provide nutrition for their hosts^1,7^. So far, most studies have focused on inorganic carbon fixation by the symbionts and the transfer of fixed organic carbon compounds to the hosts. In addition to organic carbon, all animals require a source of fixed nitrogen. However, nitrogen metabolism in chemosynthetic symbioses has received far less attention. Chemosynthetic symbionts have been shown to gain their nitrogen from ammonium or nitrate in their environment^8–10^ and co-occurring nitrogen-fixing and chemosynthetic symbionts have been found in cold-water corals^11^. Nitrogen fixation by chemosynthetic symbionts has long been hypothesized, but so far not yet shown^12–14^.

Our study focused mainly on the endosymbiosis between bivalves of the family Lucinidae and sulfur-oxidizing bacteria. Lucinids are by far the most diverse and widespread group of bivalves that host chemosynthetic symbionts^15^. There are at least 400 living species, occupying a range of habitats including mangrove sediments, seagrass beds, coral reef sediments and coastal mud and sand^16^. In seagrass habitats, lucinid bivalves and their sulfur-oxidizing symbionts are part of a nested symbiosis with seagrasses, which may be essential to the health and ecological success of seagrasses^6^. We focused on the symbiosis between *Loripes lucinalis* (Lamarck, 1818) and its endosymbionts. We also investigated a second symbiosis, that between stilbonematid nematodes and their sulfur-oxidizing ectosymbionts, because these symbionts are associated with the family Chromatiaceae, which contains a number of diazotrophic sulfur oxidizers^17,18^. Nematodes of the subfamily Stilbonematinae (family Desmodoridae) can be found worldwide in marine sulfidic habitats^19^. All known species have a dense coating of ectosymbionts on their cuticle, which are hypothesized to contribute to their host's nutrition^19^. The name *Candidatus* Thiosymbion oneisti will be proposed elsewhere for the nematode symbionts (Gruber-Vodicka *et al.*, in preparation). We propose the name *Candidatus* Thiodiazotropha endoloripes for the symbiont of *Loripes lucinalis*, where ‘Thiodiazotropha’ refers to the sulfur-oxidizing (‘thio’) and nitrogen-fixing (‘diazotroph’) metabolism of the symbiont and ‘endoloripes’ (‘Endo-’, Greek from *ἔνδον* meaning ‘within’, ‘loripes’) refers to the endosymbiotic association with *Loripes lucinalis*, its bivalve host.

## Results and discussion

### Phylogenomics, and carbon and energy metabolism of *L. lucinalis* and *Laxus oneistus* symbionts

**Figure 1:**
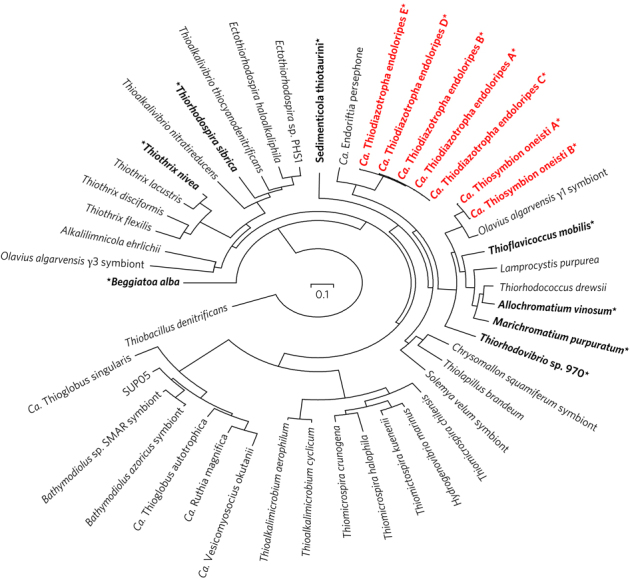
Phylogenomic tree of sulfur-oxidizing Gammaproteobacteria. Phylogenomic tree of free-living and symbiotic sulfur oxidizers from the Gammaproteobacteria. The 25 single-copy genes used in the analysis were defined based on the AMPHORA2 core bacterial phylogenetic marker database^66^. SUP05 bin refers to the genomic assembly from the metagenome study by Walsh and co-authors^81^. The betaproteobacterial sulfur oxidizer *Thiobacillus denitrificans* was used as the outgroup. SH-like support values were above 90% for all nodes of the tree. Genomes encoding nitrogenase genes are indicated with an asterisk and bold text. Sequences from this study are shown in red.

The symbiont draft genomes from five clam individuals (*Ca*. Thiodiazotropha endoloripes A–E) were 100% complete for a set of 281 marker genes conserved across all Gammaproteobacteria and ranged in size from 4.46 to 4.88 megabase pairs (Mb) on 12–48 contigs (Table 1). Those from two individuals of *L. oneistus* (*Ca*. Thiosymbion oneisti A–B) were 86.75 and 89.11% complete at sizes of 3.66 and 3.51 Mb on 183 and 193 contigs (Table 1). We conducted phylogenomic analyses to better understand the relationships between the bivalve and nematode symbionts and other symbiotic and free-living gammaproteobacterial sulfur oxidizers. Consistent with previous analyses based on 16S rRNA genes^1^, our phylogenomic analysis placed the lucinid symbionts *Ca*. Thiodiazotropha endoloripes together in a cluster with *Candidatus* Endoriftia persephone, the sulfur-oxidizing symbiont of the hydrothermal vent tubeworm *Riftia pachyptila* (Fig. 1). Also consistent with previous analyses^20^, the nematode symbionts *Ca*. Thiosymbion oneisti clustered together with the sulfur-oxidizing endosymbionts of the gutless oligochaete worm *Olavius algarvensis* and were affiliated with free-living sulfur oxidizers from the family Chromatiaceae (Fig. 1). The *L. lucinalis* and *R. pachyptila* symbiont cluster was not clearly associated with any known free-living sulfur oxidizers, but formed a sister group to the clade containing the stilbonematid and oligochaete symbionts and members of the Chromatiaceae (Fig. 1).

**Table 1:** Features of bivalve and nematode symbiont genomes.

Genome	Size (Mb)	No. of contigs	No. of genes predicted	GC content (%)	Completeness estimate* (%)
*Ca.* Thiodiazotropha endoloripes A	4.46	15	4,193	52.1	100
*Ca.* Thiodiazotropha endoloripes B	4.61	18	4,381	51.9	100
*Ca.* Thiodiazotropha endoloripes C	4.46	18	4,226	52.1	100
*Ca.* Thiodiazotropha endoloripes D	4.5	12	4,301	52.0	100
*Ca.* Thiodiazotropha endoloripes E	4.88	48	4,685	51.7	100
*Ca.* Thiosymbion oneisti A	4.44	2,026	4,149	58.71	96.63
*Ca.* Thiosymbion oneisti B	4.33	1,891	4,050	58.84	96.07

*Completeness estimates were calculated based on how many of 281 conserved gammaproteobacterial marker genes were present in each draft genome. See Methods for details.

**Figure 2:**
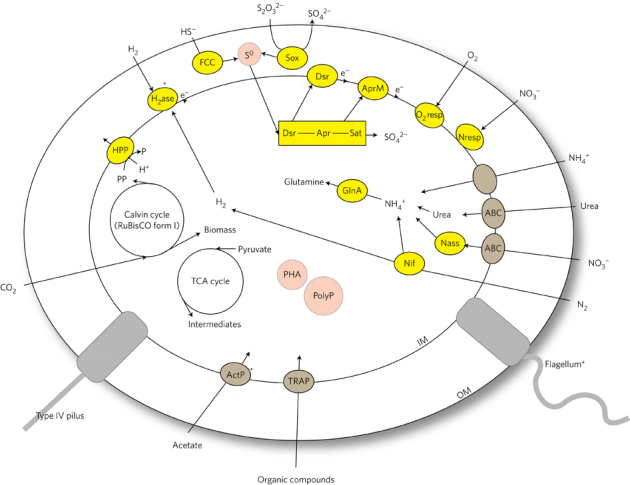
Overview of the major cellular features and metabolic pathways encoded in bivalve and nematode symbiont genomes. Metabolic enzymes and enzyme systems are shown in yellow, transporters in brown, storage granules in pink and structural features in grey. Features only encoded in the draft genomes of *Ca*. Thiodiazotropha endoloripes and not yet found in the draft genomes of *Ca*. Thiosymbion oneisti are indicated by an asterisk. IM, inner membrane; OM, outer membrane; HPP, proton-translocating pyrophosphatase; H_2_ase, uptake hydrogenase; FCC, flavocytochrome *c*; S^0^, elemental sulfur granule; Sox, sox enzyme system for sulfur oxidation; Dsr, reverse dissimilatory sulfite reductase; Apr, adenosine phosphosulfate reductase; AprM, adenosine 5′-phosphosulfate membrane anchor; Sat, sulfate adenylyltransferase; O_2_resp, genes for oxygen respiration (cytochrome *c* oxidases); Nresp, nitrate respiration (denitrification, pathway complete to N_2_); Nass, assimilatory nitrate reduction; ActP, acetate transporter; ABC, ABC transporter; TRAP, TRAP transporter; PolyP, polyphosphate granule; PHA, polyhydroxyalkanoate granule; GlnA, glutamine synthetase; Nif, nitrogenase.

As expected based on previous studies of symbiont metabolism, genes and pathways for sulfur oxidation and carbon fixation were found in all five *Ca*. Thiodiazotropha endoloripes and in both *Ca*. Thiosymbion oneisti draft symbiont genomes (Fig. 2). Nematode and bivalve symbionts encoded a complete tricarboxylic acid (TCA) cycle and transporters for uptake of organic compounds and thus have the potential for heterotrophic growth. Both are capable of using oxygen and oxidized nitrogenous compounds such as nitrate as terminal electron acceptors, but only the bivalve symbiont draft genomes encoded genes for uptake hydrogenases, which would allow them to use hydrogen as an energy source. Bivalve and nematode symbionts both produce intracellular elemental sulfur granules^21,22^. The genomes also revealed the potential to store organic carbon in the form of polyhydroxyalkanoates (PHAs) and phosphorous in the form of polyphosphate granules. A detailed comparative genomics study of the lucinid and stilbonematine symbionts is beyond the scope of this study and will be published elsewhere.

### Symbionts of *L. lucinalis* and *L. oneistus* encode nitrogenases

**Figure 3:**
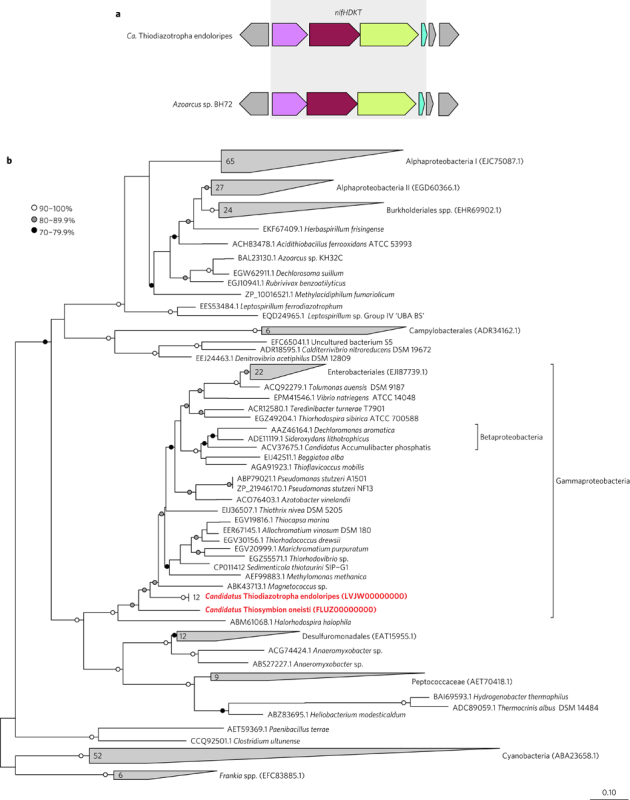
Clusters of *nif* genes and NifH phylogeny. **a**, Schematic representation of the *nifHDKT* gene cluster in the *Ca*. Thiodiazotropha endoloripes draft genomes and in the model nitrogen-fixing bacterium *Azoarcus* sp. BH72. See Supplementary Fig. 2 for an overview of the complete *nif* cluster. **b**, Maximum-likelihood phylogeny of full-length Group 1 NifH proteins. Percentages refer to SH-like support values from aLRT. Sequences from this study are shown in bold red text. Numbers in wedges indicate how many sequences are contained in that collapsed clade. Brackets contain examples of specific protein sequences.

Surprisingly, the five draft genomes of *Ca*. Thiodiazotropha endoloripes and both draft genomes of *Ca*. Thiosymbion oneisti contained large clusters of genes involved in nitrogen fixation, including the structural genes for the iron-molybdenum dinitrogenase (*nifD* and *nifK*), the dinitrogenase reductase subunit (*nifH*), ferredoxins, and maturation and regulatory factors (Fig. 3, Supplementary Discussion and Supplementary Figs 1 and 2). The *nifH* gene is commonly used as a functional marker for nitrogen fixation, so many *nifH* genes are available in public databases. Our phylogenetic analyses showed that the sulfur-oxidizing symbiont sequences clustered together with Group 1 molybdenum-dependent NifH sequences as defined by Raymond^23^ (Fig. 3). The symbiont sequences fell into a clade containing mainly Gammaproteobacteria and some Betaproteobacteria. The NifH sequences from both *Ca*. Thiodiazotropha endoloripes and *Ca*. Thiosymbion oneisti grouped separately to those from other members of the Chromatiaceae, which could indicate that this gene was acquired by horizontal gene transfer in these two symbionts. To gain more insight into the evolutionary history of nitrogen fixation in *Ca*. Thiodiazotropha endoloripes and *Ca*. Thiosymbion oneisti, we analysed the phylogeny of the NifD proteins, which make up one of the two structural subunits of the nitrogenase enzyme. The placement of NifD proteins from the chemosynthetic symbionts was different to the placement of their NifH proteins (Supplementary Fig. 3). The NifD from *Ca*. Thiodiazotropha endoloripes grouped together with the NifD from *Sedimenticola thiotaurini* (Supplementary Fig. 3). This was similar to our phylogenomic results, which showed that *S. thiotaurini* grouped together with the Chromatiaceae (Fig. 1). The NifD of *Ca*. Thiosymbion oneisti grouped together with the NifD from *Methylomonas methanica* and not with those from other members of the Chromatiaceae (Supplementary Fig. 3). In summary, there appears to be a history of horizontal transfer of the genes encoding both NifH and NifD, so these proteins could have been horizontally acquired by the chemosynthetic symbionts of lucinid clams and stilbonematid nematodes.

Using PCR primers specific for *nifH*, we screened DNA extracted from six *L. lucinalis* individuals, three from France and three from Mauritania, and DNA from five additional lucinid species from sampling sites around the world (Supplementary Tables 1 and 2). We obtained bands of the correct size from all individuals of *L. lucinalis* and from three other lucinid species (*Euanodontia ovum*, *Codakia orbicularis* and *Clathrolucina costata*) (Supplementary Table 2). We could not obtain a PCR product from one individual each of *Lucinoma borealis* and *Epidulcina cf delphinae*, although the symbiont 16S rRNA gene could be amplified from these samples (Supplementary Table 2). The PCR products were directly sequenced and, although they were too short to determine their phylogenetic position reliably, they were highly similar to the sequences we obtained from the symbiont genomes. All *nifH* sequences from *L. lucinalis* symbionts were between 92 and 100% identical at the nucleotide level (97–100% identity at the amino acid level). Among symbionts of different lucinid species, the *nifH* sequence identity ranged from 83 to 88% (91–98% at the amino acid level). Genome sequencing of these symbiont species would confirm whether they also encode all genes necessary for nitrogen fixation. However, the presence of the *nifH* gene raises the possibility that the symbionts of many lucinid species might be capable of nitrogen fixation.

Nitrogen-fixing symbioses are common in marine ecosystems, particularly in habitats where nitrogen availability limits primary production, such as oligotrophic coral reefs and open ocean water^24,25^. Chemosynthetic symbionts are well known for their contribution to host nutrition through carbon fixation, but so far, no marine chemosynthetic symbiont was known to be capable of nitrogen fixation. Nitrogen fixation has been hypothesized in the only known chemosynthetic symbiosis in the terrestrial environment between ectosymbiotic *Thiothrix*-related bacteria and *Niphargus* amphipods, because *nifH* transcripts could be PCR-amplified from the *Niphargus* ectosymbiotic community^26^. In contrast to previous PCR-based studies, we could unambiguously associate the *nifH* sequence with the rest of the symbiont genome. The presence of these genes in the symbionts of nematodes and bivalves, two unrelated hosts from different animal phyla, shows that the ability to fix nitrogen is not restricted to one phylogenetic group of symbionts, or to the symbionts of only one animal group, but may be widespread in chemosynthetic symbioses.

### Nitrogen fixation genes are expressed by *L. lucinalis* symbionts

**Figure 4:**
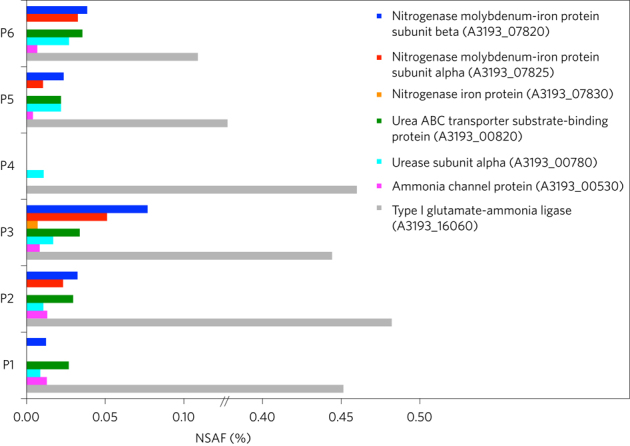
Expression of proteins for nitrogen assimilation in *Loripes lucinalis.* Bar chart showing the abundance of seven symbiont proteins involved in assimilation of nitrogen from urea, ammonia and dinitrogen gas that were identified by proteomics in the gills of six *L. lucinalis* individuals (P1–P6). See Supplementary Data set 2 for the complete data set. NSAF, normalized spectral abundance factor.

To test whether nitrogen fixation genes are actively expressed by the lucinid symbionts when living in their hosts, we sequenced the gill metatranscriptomes of five individuals and analysed gill metaproteomes of another six individuals of *L. lucinalis* from Elba (Italy). Transcripts from genes involved in nitrogen fixation were among the 30 most abundantly expressed genes in two of these five individuals (Supplementary Fig. 4 and Supplementary Data set 1). In gill metaproteomes from six *L. lucinalis* individuals, between 892 and 1,377 symbiont proteins could be detected (Supplementary Data set 2). Nitrogenase proteins were detected in five of these six individuals (Fig. 4 and Supplementary Data set 2). Nitrogen fixation is therefore one of the metabolic pathways actively expressed by the symbionts in some *L. lucinalis* individuals (see Supplementary Discussion for further details).

It is remarkable that the symbionts in the animal tissue actively express nitrogenases and seem to be nitrogen-limited (Supplementary Discussion). Because nitrogen fixation is metabolically costly, it is often downregulated when other nitrogen sources are available in the environment^27,28^. As far as we are aware, the concentrations of nitrate, ammonium and urea have not yet been measured in lucinid tissues. Animals are known to produce ammonia and urea as nitrogenous waste products and both of these could be used as nitrogen sources by the lucinid and stilbonematid symbionts (Fig. 2). It is possible that symbiont nitrogen fixation is regulated by the availability of these nitrogen sources in the gill tissues, or in the immediate environment of the bivalves, similar to the scenario in tropical corals, where primary productivity by zooxanthellae benefits from fixed nitrogen when other nutrient sources are lacking^29^. It is interesting to note the parallel between carbon and nitrogen fixation in lucinid symbiosis. The symbionts clearly also fix carbon inside the animal tissue, although the environment experienced by the symbionts inside the host is presumably replete with organic compounds and the symbionts have the capability to grow heterotrophically. In the future, it would be intriguing to investigate how the presence of alternative carbon and nitrogen sources influences the regulation of nitrogen and carbon fixation by the symbionts.

### Signature of nitrogen fixation in *L. lucinalis* stable isotope ratios

Stable isotopes provide a valuable record of the nutrition sources used by organisms in their natural environment. The stable nitrogen and carbon isotopic composition of the biomass of primary producers is usually lighter than their inorganic carbon and nitrogen source due to the slight preference enzymes such as the ribulose-1,5-bisphosphate carboxylase/oxygenase (RuBisCO) and nitrogenase have for the lighter isotope^30^. This shift is called fractionation. In consumers, fractionation leads to enrichment of ^13^C and ^15^N because of the preferential release of the lighter isotopes and induces a shift in the remaining biomass to heavier values^31^. Newly fixed nitrogen typically has a δ^15^N between −2 and 0‰ (ref. 30). This is very similar to the composition of atmospheric nitrogen gas (by definition δ^15^N = 0‰), because fractionation during microbial nitrogen fixation is minimal^30^. In contrast, values for heterotrophic marine filter-feeding bivalves are typically larger than 6‰ because they feed on material that is enriched in ^15^N (ref. 32). We analysed the stable carbon and nitrogen isotopic composition of *L. lucinalis* and three symbiont-free bivalve species, *Senilia senilis*, *Pelecyora isocardia* and *Diplodonta circularis* (see Supplementary Table 3 for an overview of stable isotope data). These species co-occur with *L. lucinalis* in intertidal flats of the Banc d'Arguin, Mauritania, and thus presumably have access to the same nutrient sources in their environment^33^. For comparison, we also analysed the stable carbon and nitrogen isotopic composition of 20 *L. lucinalis* individuals from Elba (Supplementary Table 3).

**Figure 5:**
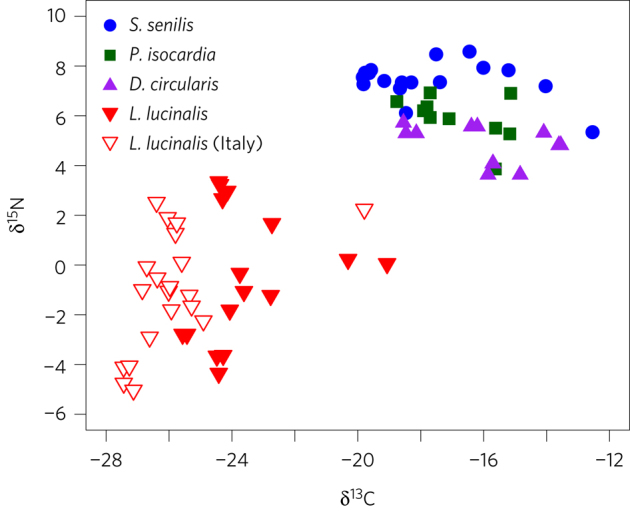
Carbon and nitrogen stable isotope ratios (‰) of symbiont-free and symbiont-hosting bivalves. Both δ^13^C and δ^15^N signatures (‰) of *L. lucinalis* (*N* = 13) are significantly more depleted compared to three symbiont-free bivalve species *S. senilis* (*N* = 17), *P. isocardia* (*N* = 10) and *D. circularis* (*N* = 5) found on the intertidal flats of Banc d'Arguin, Mauritania. See Supplementary Table 4 for parameter estimates of the models, with carbon and nitrogen isotope signature (‰) modelled as a function of species. All samples were collected at Banc d'Arguin, Mauritania, unless otherwise specified.

The model including an effect of species was significantly better than the intercept model for both carbon (log ratio statistic = 72.83, degrees of freedom = 4, *P* < 0.0001) and nitrogen stable isotope ratios (log ratio statistic = 98.89, degrees of freedom = 4, *P* < 0.0001). As expected, the δ^13^C values of *L. lucinalis* (δ^13^C (mean ± s.d.) = −23.4 ± 1.8‰) were significantly more depleted than those of the three symbiont-free bivalve species *S. senilis* (δ^13^C = −17.7 ± 2.2‰), *P. isocardia* (δ^13^C = −16.9 ± 1.3‰) and *D. circularis* (δ^13^C = −16.4 ± 2.0‰), consistent with a chemosynthetic source of carbon for *L. lucinalis* (Fig. 5 and Supplementary Table 4). The δ^13^C values of *L. lucinalis* from Elba (δ^13^C = −25.9 ± 1.6‰) were significantly more depleted than those collected at Banc d'Arguin (−2.6, standard error (SE) 1.0, *P *= 0.01; Fig. 5 and Supplementary Table 4). *L. lucinalis* can also filter-feed and can vary the relative proportion of nutrition it gains from heterotrophic feeding depending on the environmental conditions^34^. The difference in δ^13^C signatures between Banc d'Arguin and Elba suggests that *L. lucinalis* from the subtidal Elba habitat rely on their endosymbionts for a larger proportion of their carbon nutrition than those from the intertidal Banc d'Arguin, although this difference could also be due to the different handling of samples from Italy and Mauritania, as those from Italy were frozen immediately after sampling and those from Mauritania were first kept in aquaria for 24 h.

The δ^15^N values of *L. lucinalis* (δ^15^N (mean ± s.d.) = −0.5 ± 2.8‰) were also significantly more depleted than those of the three symbiont-free species species *S. senilis* (δ^15^N = 7.4 ± 0.8‰), *P. isocardia* (δ^15^N = 5.9 ± 0.9‰) and *D. circularis* (δ^15^N = 5.1 ± 0.7‰) (Fig. 5 and Supplementary Table 4). The δ^15^N values of *L. lucinalis* from Banc d'Arguin and those from Elba (δ^15^N = −1.1 ± 2.3 ‰) were not significantly different (−0.61, SE = 1.03, *P *= 0.56, Supplementary Table 4). The mean δ^15^N value of *L. lucinalis* (Banc d'Arguin, −0.5‰; Elba, −1.1‰) fell exactly in the range expected for newly fixed nitrogen. This is consistent with active nitrogen fixation by the symbionts, a result that should be corroborated in future studies, for example by isotope tracer experiments.

The δ^15^N values for *L. lucinalis* from both Mauritania and Italy varied remarkably between individuals, and ranged from −4.4‰ to +3.3‰ (Mauritania) and from −5.0‰ to +2.5‰ (Elba) (Fig. 5). This is intriguing considering our transcriptomics and proteomics results, which showed that nitrogenases were not detectable in all *L. lucinalis* individuals analysed. The variability in δ^15^N signatures between individuals might reflect differences in the relative contribution of nitrogen fixation, the uptake of alternative nitrogen sources such as ammonium, urea or nitrate by the lucinid symbionts, and filter feeding by the host.

### Concurrent aerobic and anaerobic metabolism in *L. lucinalis* symbiont populations

All known nitrogenase enzymes are extremely sensitive to oxygen^35^. The bivalve gill, which is the site for aerobic gas exchange in the animal, therefore seems to be an unusual location for nitrogen-fixing bacteria, but this is not unprecedented—the nitrogen-fixing symbionts of wood-boring bivalves are housed in the host's gill^36^. The symbionts of wood-boring bivalves have been clearly shown to fix nitrogen when inside the gill^37^. Oxygen respiration by bacteria requires terminal cytochrome oxidases, of which a number of different types are known. For example, aa3 type terminal oxidases function best under atmospheric oxygen concentrations, while cbb3 type terminal oxidases function best under reduced oxygen concentrations^38^. *Bradyrhizobium japonicum*, the nitrogen-fixing symbiont of leguminous plants, switches to expressing a cbb3 type terminal oxidase to adapt to the low oxygen concentrations found in the root nodules of plants^39^. The plant host expresses leghaemoglobins that bind oxygen, keeping concentrations low in root nodules and preventing damage to the nitrogenases expressed by their nitrogen-fixing symbionts^40^. *L. lucinalis* is not known to produce haemoglobins, but they have been reported in other lucinid species^41^. *Ca*. Thiodiazotropha endoloripes encodes both the low-affinity aa3 and the high-affinity cbb3 type oxidases. Transcripts for the aa3 type cytochrome oxidase were among the 30 most abundant in all transcriptomes and were also detected in all six proteomes, suggesting that at least some symbionts experience relatively high oxygen concentrations (Supplementary Fig. 4 and Supplementary Data sets 1 and 2). The cbb3 type was also expressed by the symbionts in all five bivalves analysed for transcriptomics, and all six proteomes (Supplementary Data sets 1 and 2). It is also intriguing that a gene similar to anaerobic dimethyl sulfoxide reductases was one of the most highly expressed genes in all five transcriptomes and all six proteomes. Terminal molybdopterin oxidoreductases such as dimethyl sulfoxide reductases allow anaerobic respiration using terminal electron acceptors other than oxygen, in this case, possibly dimethyl sulfoxide (DMSO)^42^. Aerobic and anaerobic metabolism might therefore coexist in a single host individual, possibly by different symbiont subpopulations (Supplementary Discussion). Sub-oxic or anoxic niches in the gill tissue would provide ideal conditions for nitrogen-fixing symbionts. Alternatively, the bivalve host may control the oxygen concentrations experienced in the gill, temporally, by controlling the rate at which it pumps oxygenated sea water through its burrow. Finally, in the stilbonematid nematodes, the host's migratory behaviour would allow its symbionts to experience both oxic conditions in shallow sediment layers and anoxic conditions in the deeper sediment^43^. It is therefore possible that the nematode ectosymbionts fix nitrogen when the nematodes inhabit deep anoxic sediment layers.

### Potential roles of nitrogen fixation for the symbiosis and the ecosystem

The discovery of nitrogen-fixing chemosynthetic symbionts was at first glance surprising, considering that marine animals produce ammonium and urea as nitrogenous waste products^44^ and many chemosynthetic symbionts including those from *Ca*. Thiodiazotropha endoloripes and *Ca*. Thiosymbion oneisti have the genetic potential to use ammonium or urea as nitrogen sources (Fig. 2). Indeed, in the *Olavius algarvensis* (oligochaete annelid) chemosynthetic symbiosis, recycling of host nitrogenous waste products by the symbiotic bacteria may have resulted in the reduction of the host nephridia, the organs responsible for processing nitrogenous waste^3^. Although the recycling of host nitrogenous waste would undoubtedly result in an overall more efficient nitrogen use by the symbiosis, an external source of nitrogen would still be needed for net growth^45^. Just as carbon fixation by chemosynthetic symbionts provides a source of carbon for host nutrition, nitrogen fixation by chemosynthetic symbionts may also provide a source of nitrogen for their host.

Chemosynthetic symbioses are often found in oligotrophic habitats such as coral reef sands or sediments associated with seagrass meadows. Presumably, animal hosts living in such oligotrophic environments would benefit most from hosting nitrogen-fixing chemosynthetic symbionts. Although we do not currently know the concentration of nutrients at our sampling sites, inorganic nitrogen concentrations in seagrass ecosystems are typically one to two orders of magnitude higher in pore waters (∼1–180 µM) than in the overlying water column^46^. However, in a chemosynthetic symbiosis, where carbon is rapidly fixed, organic carbon can be in excess in relation to the demand of the autotrophic microbial community^47^. Consequently, even if other sources of nitrogen are available in their environment, the symbiont population may be nitrogen-limited and thus benefit from nitrogen fixation.

Following on from this, nitrogen fixation by chemosynthetic symbionts could contribute a source of new nitrogen to the ecosystems they inhabit. Intriguingly, one study of seagrass sediment biogeochemistry showed that porewater ammonium concentrations were higher when lucinid bivalves were present^48^. Seagrasses have been shown to grow better in the presence of lucinids, which was hypothesized to be due to the removal of toxic sulfide by their symbionts^6^. Our results point to another possible mechanism for the promotion of seagrass growth: the provision of fixed nitrogen to the porewater of the sediments, where the seagrass roots could take it up. Seagrasses clearly benefit from active plant-associated and free-living diazotrophic microbial communities that provide them with a source of fixed nitrogen^24,49^. If lucinid bivalves and their symbionts are providing a net source of nitrogen to their environments, then they might also contribute to the ecosystem nitrogen budget in habitats where they are abundant.

## Methods

### Sample collection

Lucinid bivalves of the species *L. lucinalis* were collected in the Bay of Fetovaia, Elba, Italy, for genome and transcriptome sequencing, proteomics and stable isotope analysis (see Supplementary Table 1 for more information on sampling). The bivalves were found 10–50 cm deep in sediment 4–10 m away from a seagrass meadow of *Posidonia oceanica*. Samples for DNA sequencing were preserved in RNAlater (Thermo Fisher Scientific). Samples for transcriptome sequencing were removed from the shells and placed in RNAlater immediately after sampling and transport to the field station, which took no longer than 45 min. Proteomics samples were transported to a boat on the surface in a plastic container immediately after being removed from the sediment. Once on the boat, the clams were frozen immediately on dry ice and stored on dry ice or at −80 °C or colder until protein extraction. The time from collection from the sediment to freezing was no more than 10 min. The shell length (mm) of bivalves for stable isotope analysis was measured using calipers, then the flesh was removed from the shell and frozen at −20 °C.

*Loripes lucinalis* and three co-occurring non-symbiotic (that is, heterotrophic) bivalve species (*Senilia senilis* (Linnaeus, 1758), *Pelecyora isocardia* (Dunker, 1845) and *Diplodonta circularis* (Dunker, 1846)) were collected from the intertidal flats surrounding Iwik Peninsula, Banc d'Arguin, Mauritania, for stable isotope analyses (Supplementary Table 1). A sediment core (15 cm diameter, 20 cm depth) was taken at 40 different sampling sites, some bare, some covered with *Zostera noltii* seagrasses, and sieved over a 1 mm mesh. The 40 sites were spread over an area of ∼36 km^2^. From each benthic sample, bivalves were sorted within 12 h at the field station in Iwik. The shell length (mm) of each bivalve was measured with calipers and one individual per species was placed in an aquarium with 0.7-µm-filtered, oxygenated sea water to clear their gut contents. Animals at least 4 mm in length were chosen to provide enough biomass for carbon and nitrogen isotope measurements. After 24 h, bivalves were taken out of the aquarium, then the flesh was removed from the shell and stored frozen at −20 °C.

*L. lucinalis* for PCR amplification of *nifH* and 16S rRNA genes were collected from Banc d'Arguin, Mauritania, and Thau Lagoon, France (Supplementary Table 1). In Mauritania, bivalves were sieved from seagrass sediments and roots, and the shells were immediately opened before the bivalves were stored in RNAlater at −20 °C. In France, *L. lucinalis* were sorted from sediment within 10 min after collection and immediately fixed in 96% ethanol. Bivalves were stored at room temperature until DNA extraction.

*Codakia orbicularis* (Linneaus, 1758) was collected from St George's Cay, Bahamas, under permit from the Bahamas Government (N. Higgs, MAMR&LG/FIS/17). Gill tissues were dissected and fixed in RNAlater shortly after collection, then stored at −20 °C.

*Euanodontia ovum* (Reeve, 1850) was collected from Mauritius, and *Clathrolucina costata* (d'Orbigny, 1842) was collected from Curaçao (Supplementary Table 1). Both species were sampled by digging in seagrass beds with a shovel and trowels. Seagrass rhizomes and root masses were sieved through 2 mm mesh sieves. After collection, the bivalves were placed in cool bags then later slightly opened in the laboratory with a scalpel blade and preserved in 100% ethanol. The ethanol was changed after one day. Samples were stored at room temperature until processing.

*Laxus oneistus* was collected from Carrie Bow Cay, Belize. Nematodes were extracted from the sand by shaking in sea water and pouring the supernatant through a mesh screen with a pore size of 63 µm. Single individuals were then picked by hand under a dissecting microscope then stored in RNAlater.

### DNA extraction, genome sequencing and analysis

DNA for metagenome sequencing was extracted from homogenates from a single whole gill from each of five *L. lucinalis* from Elba (*L. lucinalis* A–E) according to the method of Zhou and colleagues^50^. DNA was extracted from two whole *L. oneistus* individuals (*L. oneistus* A and B) with intact symbiotic coat using the Blood and Tissue Micro Kit (Qiagen) according to the manufacturer's instructions. After extraction, DNA concentration was measured with the fluorometric quantitation tool Qubit (Life Technologies). From each lucinid individual, 1 µg DNA was used for PE-library preparations using the NEBNext kit (New England Biolabs). For each stilbonematid individual, 5 ng DNA was used with an Ovation Ultralow Library Systems kit (NuGEN). All libraries were size-selected on an agarose gel and sequenced at the Max Planck Genome Centre in Cologne, Germany. Illumina HiSeq 2 × 100 bp paired-end reads were sequenced for each lucinid individual (30 million reads each) and each stilbonematid (18 million reads each). The assembly for all samples was done with SPAdes 3.1 (ref. 51) after removing adapters and low-quality reads with bbduk (https://sourceforge.net/projects/bbmap/) by setting a minimum quality value of two and a minimum length of 36. Single reads were excluded from the analysis. To largely remove the host genomic reads for the lucinid samples, the reads were split based on a kmer frequency analysis performed with bbnorm. Only reads with average kmer frequencies of 30 for *L. lucinalis* A, B and D, 25 for *L. lucinalis* C, 50 for *L. lucinalis* E and 25 for *L. oneistus* A and B were kept using bbnorm (https://sourceforge.net/projects/bbmap/). The assembly was carried out using kmers 21, 33, 55 and 77. The initial binning was done using Metawatt 2.1 (ref. 52). Final binning was done interactively by collecting all contigs linked to this initial high confidence bin using the FASTG linkage information provided from the SPAdes assembly program with gbtools version 2.4.4 (ref. 53). We binned a draft symbiont genome from each of five individuals of *L. lucinalis*. No other bacterial genomes were identified in our five metagenomic libraries. The genome completeness for all samples was calculated using checkM version 1.05 (ref. 54) and the gammaproteobacterial marker gene set using the taxonomy workflow. The annotation was performed using RAST (ref. 55).

For PCR amplification of 16S rRNA and *nifH* genes from diverse lucinid species, DNA was extracted with a DNeasy Blood and Tissue kit from Qiagen according to the manufacturer's instructions.

### RNA extraction, transcriptome sequencing and analysis

DNA and RNA were co-extracted from one whole gill of five *L. lucinalis* individuals from the Bay of Fetovaia, Elba (different from those used for genome sequencing), using an AllPrep DNA/RNA mini kit according to the manufacturer's instructions (Qiagen). From each sample, at least 10 ng RNA were used for paired-end library preparations. Total RNA was converted to double-stranded cDNA and amplified linearly with the Ovation RNA-Seq System V2 kit (NuGEN). Then, 300 ng double-stranded cDNA was fragmented to an average size of 400 bp (Covaris). Illumina-compatible libraries were generated with the NEBNext Ultra DNA Library Prep Kit for Illumina kit (NEB). Finally, fragments were enriched by a PCR step for six cycles. Quality assessment was carried out at various steps at the RNA or DNA level with an Agilent Bioanalyser. DNA was quantified by fluorometry (Qubit, Thermo Fisher Scientific). Libraries were quantified by fluorometry, immobilized and processed onto a flow cell with a cBot (Illumina), followed by sequencing with TruSeq v3 chemistry on a HiSeq2500 at the Max Planck Genome Centre in Cologne, Germany. All libraries were sequenced multiplexed on single lanes of two consecutive runs that yielded a total of 26 million Illumina HiSeq 2 × 100 bp paired-end reads per individual. Paired reads generated from mRNA transcripts were indexed and aligned to genes predicted from annotation of *Ca*. Thiodiazotropha endoloripes A using bwa version 0.7.12-r1039 (index, aln and sampe) with default parameters^56^. We mapped the transcriptome reads from each individual sequenced to all five symbiont draft genomes (generated from another five individual clams). Approximately 0.5% of the transcriptome reads from each individual mapped to symbiont protein-coding genes (Supplementary Fig. 5). We plotted the identity (%) of each transcriptome read mapped to each genome. This analysis showed similar patterns for *Ca.* Thiodiazotropha endoloripes A, B, C and D, with the majority of mapped reads being 100% identical to the genome to which they were mapped, but the identities to E were generally lower (Supplementary Fig. 5). These results may indicate that *Ca*. Thiodiazotropha endoloripes A, B, C and D and all five symbiont populations used for transcriptome sequencing were highly similar and *Ca*. Thiodiazotropha endoloripes E was by chance a slightly different strain. We therefore continued the transcriptome analysis with the results of mapping to one representative symbiont genome, *Ca*. Thiodiazotropha endoloripes A. Mapping files were sorted and the number of properly paired mapped reads specific to each gene was extracted with Samtools view (-f 3)^57^. To rank the genes detected in the transcriptome of each individual according to the abundance of their transcripts, we calculated the number of transcripts that mapped to each gene as a percentage of the total number of transcripts that mapped to all genes (‘% total counts’). We then adjusted this percentage according to gene length and ranked the genes according to the percentage of all transcripts that mapped per kb per gene (‘% of total counts adjusted to gene length’).

### Protein extraction and analysis

We prepared tryptic digests from six biological replicates (one whole gill from each of six individuals frozen immediately after sampling, see section ‘Sample collection’) following the filter-aided sample preparation (FASP) protocol described by Wiśniewski *et al*.^58^ with some small modifications as described by Hamann and co-authors^59^. Peptides were desalted using Sep-Pak C18 Plus Light Cartridges (Waters) according to the manufacturer's instructions. Approximate peptide concentrations were determined using the Pierce Micro BCA assay (Thermo Scientific Pierce) following the manufacturer's instructions.

Samples were analysed by one-dimensional liquid chromatography tandem mass spectrometry. For each sample a technical replicate was run. Two blank runs were done between samples to reduce carry over. For each run, 2,000 ng of peptide were loaded onto a 2 cm, 75 µm ID C18 Acclaim PepMap 100 pre-column (Thermo Fisher Scientific) using an EASY-nLC 1000 liquid chromatograph (Thermo Fisher Scientific) set up in two-column mode. The pre-column was connected to a 50 cm × 75 µm analytical EASY-Spray column packed with PepMap RSLC C18, 2 µm material (Thermo Fisher Scientific), which was heated to 35 °C using the integrated heating module. The analytical column was connected via an Easy-Spray source to a Q Exactive Plus hybrid quadrupole-Orbitrap mass spectrometer (Thermo Fisher Scientific). Peptides were separated on the analytical column at a flow rate of 225 nl min^–1^ using a 460 min gradient going from buffer A (0.2% formic acid, 5% acetonitrile) to 20% buffer B (0.2% formic acid in acetonitrile) in 354 min, then from 20 to 35% B in 71 min and ending with 35 min at 100% B. Eluting peptides were ionized with electrospray ionization (ESI) and analysed in the Q Exactive Plus. Full scans were acquired in the Orbitrap at 70,000 resolution. MS/MS scans of the 15 most abundant precursor ions were acquired in the Orbitrap at 17,500 resolution. The mass (*m*/*z*) 445.12003 was used as lock mass as described by Olsen *et al*.^60^ with the modification that lock mass was detected in the full scan rather than by separate SIM scan injection. Lock mass use was set to ‘best’. Ions with charge state +1 were excluded from MS/MS analysis. Dynamic exclusion was set to 30 s. Roughly 500,000 MS/MS spectra were acquired per sample (two technical replicates combined).

For protein identification, a database was created using all protein sequences predicted from the *Ca.* Thiodiazotropha endoloripes genomes published in this study. CD-HIT was used to remove redundant sequences from the database^61^. The cRAP protein sequence database (http://www.thegpm.org/crap/) containing protein sequences of common laboratory contaminants was appended to the database. The final database contained 6,816 protein sequences. The database was submitted to the PRIDE repository (see section ‘Data availability’). For protein identification, MS/MS spectra were searched against the database using the Sequest HT node in Proteome Discoverer version 2.0.0.802 (Thermo Fisher Scientific) with the following parameters: Trypsin (Full), max. 2 missed cleavages, 10 ppm precursor mass tolerance, 0.1 Da fragment mass tolerance and max. 3 equal dynamic modifications per peptide. The following three dynamic modifications were considered: oxidation on M (+15.995 Da), carbamidomethyl on C (+57.021 Da) and acetyl on the protein N terminus (+42.011 Da). False discovery rates (FDRs) for peptide spectral matches (PSMs) were calculated and filtered using the Percolator Node in Proteome Discoverer. The Percolator algorithm ‘uses semi-supervised learning and a decoy database search strategy to learn to distinguish between correct and incorrect PSMs’^62^. Percolator was run with the following settings: maximum Delta Cn 0.05, a strict target FDR of 0.01, a relaxed target FDR of 0.05 and validation based on *q*-value. The Protein FDR Validator Node in Proteome Discoverer was used to classify protein identifications based on *q*-value. Proteins with a *q*-value of <0.01 were classified as high-confidence identifications and proteins with a *q*-value of 0.01–0.05 were classified as medium-confidence identifications. Only proteins identified with medium or high confidence were retained, resulting in an overall FDR of 5%. Based on these filtering criteria, between 892 and 1,377 proteins were identified per sample. The sample reports were then exported as a tab-delimited file for further processing.

For protein quantification, normalized spectral abundance factors (NSAFs) were calculated based on the number of PSMs per protein using the method described by Florens *et al*.^63^ and multiplied by 100. The NSAFx100 gives the relative abundance of a protein in a sample in %.

### PCR amplification and sequencing of nifH and 16S rRNA genes

DNA extracted from diverse lucinid species was used as a template for amplification using 16S rRNA- and *nifH*-specific primers and the DreamTaq DNA Polymerase (Thermo Fisher Scientific). Primers 27F and 1492R were used to amplify the 16S rRNA gene^64^. Degenerate primers IGK3/DVV were used to amplify the *nifH* gene^65^. The reaction conditions were as follows: one cycle at 94 °C (4 min); 32 cycles and 34 cycles (for the 16S rRNA and *nifH* genes, respectively) at 94 °C (30 s), 52 °C (45 s) and 72 °C (45 s); plus one final cycle at 72 °C (10 min). The resulting PCR products were purified with a QIAquick PCR Purification Kit from Qiagen and Sanger sequenced by Microsynth AG.

### Phylogenomic analysis

Available genomes of gammaproteobacterial sulfur oxidizers were retrieved from GenBank and the Joint Genome Institute's Integrated Microbial Genomes database (JGI-IMG). Phylogenomic treeing of these genomes along with the lucinid and stilbonematid symbiont genomes was performed using scripts that were available at phylogenomics-tools (doi:10.5281/zenodo.46122). Marker proteins that are universally conserved across the bacterial domain were extracted from the sulfur-oxidizer genomes using the Amphora2 pipeline^66^. Twenty-five markers (*frr*, *infC*, *nusA*, *pgk*, *pyrG*, *rplB*, *rplC*, *rplD*, *rplE*, *rplF*, *rplM*, *rplN*, *rplP*, *rplS*, *rplT*, *rpmA*, *rpsB*, *rpsC*, *rpsE*, *rpsI*, *rpsJ*, *rpsK*, *rpsM*, *rpsS* and *tsf*) that were identified to be occurring in single copy in all of the genomes were used for alignment on Muscle^67^. An alignment mask was generated using Zorro^68^. Poorly aligned regions or misaligned regions were visually identified and removed from the alignments. The marker alignments were further concatenated into a single partitioned alignment and the best protein substitution model for each of the markers (*frr*, *nusA*, *pyrG*, *rplB*, *rplC*, *rplD*, *rplE*, *rplF*, *rplM*, *rplP*, *rplS*, *rplT*, *rpsB*, *rpsC*, *rpsM*, *tsf*: LG; *infC*, *rpsE*, *rpsJ*, *rpsS*: JTT; *pgk*, *rpmA*: WAG; *rplN*, *rpsK*: RTREV; *rpsI*: JTTDCMUT) was predicted using the concat_align.pl script (phylogenomics-tools). The best tree with SH-like aLRT support values^69^ was finally defined on RAxML (ref. 70) using the tree_calculations.pl script on phylogenomics-tools.

### Phylogenetic analysis of NifH and NifD

Symbiont *nifH* and *nifD* gene sequences were obtained from the RAST annotations. Gene sequences from *S. thiotaurini* SIP-G1 (ref. 71) were downloaded from GenBank (CP011412). Sequences were imported using ARB^72^ into curated databases for *nifH* (http://www.zehr.pmc.ucsc.edu/nifH_Database_Public/)^73^ and *nifD* (http://www.css.cornell.edu/faculty/buckley/nifh.htm^74^) respectively and approximate placements were found with the parsimony quick-add feature in ARB. Group I *nifH* sequences were exported and dereplicated at 95% identity with Usearch 8.1 cluster-fast algorithm (length-sorted)^75^. *NifD* sequences were exported and those without nitrogenase alpha chain domains (TIGR01284, TIGR01862) were excluded as likely false positives. For both genes, the amino acid translation was aligned with Muscle 3.7 (ref. 67) and the amino acid alignment was used to guide the nucleotide alignment with TranslatorX (ref. 76). Maximum likelihood trees of amino acid and nucleotide (first and second codon positions only) alignments were calculated with PhyML 3.1 (ref. 77) using the WAG + Gamma and GTR + Gamma models, respectively, both with four Gamma rate categories and SH-like aLRT support values^69^.

### Nitrogen and carbon stable isotope analysis

Whole individuals were used for stable isotope analysis, so different individuals were used for molecular and stable isotope analysis. Tissue samples of *L. lucinalis* (*N* = 20; shell length (mean ± s.d.) = 9.0 ± 3.3 mm) from Elba (Italy) were freeze-dried for 72 h, ground to a fine powder and acidified in an HCl atmosphere to remove traces of carbonate. Dried tissues were weighed before stable isotope analysis on a continuous-flow elemental analyser-isotope ratio mass spectrometer (EA-IRMS) consisting of an elemental analyser (EA 1110, CE Instruments) coupled via a ConFlo III interface (Finnigan MAT, Thermo Fisher) to the IRMS (DeltaPLUS, Finnigan MAT, Thermo Fisher).

Tissue samples of *L. lucinalis* (*N* = 12; shell length (mean ± s.d.) = 8.2 ± 1.7 mm), *S. senilis* (*N* = 17; shell length = 40.3 ± 16.9 mm), *P. isocardia* (*N* = 10; shell length = 11.0 ± 4.8 mm) and *D. circularis* (*N* = 5; shell length = 12.0 ± 5.4 mm) collected at Banc d'Arguin, Mauritania, were freeze-dried for 72 h, homogenized by mortar and pestle, weighed into tin cups. Their carbon and nitrogen stable isotope ratios were measured with a Thermo Scientific (Flash 2000) elemental analyser coupled to a Delta V isotope mass spectrometer as described above. Tissue samples from Banc d'Arguin were not acidified before stable isotope analysis, as previous studies showed no effect of tissue treatment with HCl on δ^13^C values of *L. lucinalis*^34^.

Carbon and nitrogen stable isotope ratios (‰) are expressed in delta notation as δ^13^C or δ^15^N = (*R*_sample_/ *R*_ref_ − 1) × 1,000. *R*_sample_ is the ratio of the heavy to light isotope (^13^C/^12^C or ^15^N/^14^N) in the sample and *R*_ref_ is the same ratio for the reference material, the Vienna Pee Dee Belemnite standard for C (*R*_ref_ = 0.01118) and atmospheric nitrogen for N (*R*_ref_ = 0.00368).

### Statistical analysis of stable isotope ratios

To analyse the effect of bivalve species on carbon and nitrogen stable isotope ratios, we used linear mixed-effects models, with random intercepts and with sampling site as a random effect and species as explanatory variable. On six occasions, an individual from two different focal species was collected from the same sampling site at Banc d'Arguin (Mauritania), while multiple individuals of *L. lucinalis* were collected from the same sampling site at Elba (Italy). Assumptions of normality and homogeneity of residuals were visually inspected by plotting quantile–quantile plots and by plotting the standardized residuals versus fitted values and the standardized residuals per species. This revealed species-dependent spread in the error variances in the model with nitrogen stable isotope ratios (‰) as dependent variable, for which we accounted by adding a ‘varIdent’ variance structure to the model^78^. We tested for the significance of the species effect on stable carbon and nitrogen isotope ratios using likelihood ratio tests. Reported *P* values are for a two-tailed test. All analyses were performed in program R (R Development Core Team 2015, version 3.2.2; http://www.R-project.org). For linear mixed-effects models, the R-package nlme was used^79^.

### Data availability

Genome and transcriptome data from *L. lucinalis* have been submitted to NCBI in BioProject PRJNA314435. The draft genome sequences from lucinid symbionts are available under accession nos. LVJW00000000 (*Ca*. Thiodiazotropha endoloripes A), LVJX00000000 (*Ca*. Thiodiazotropha endoloripes B), LVJY00000000 (*Ca*. Thiodiazotropha endoloripes C), LVJZ00000000 (*Ca*. Thiodiazotropha endoloripes D) and LVKA00000000 (*Ca*. Thiodiazotropha endoloripes E). Transcriptome sequences can be found at the NCBI Short Read Archive under accession no. SRP073135. Draft genome sequences from stilbonematid nematodes have been submitted to ENA under project no. PRJEB14785 and can be found under accession nos. FLUZ00000000 (*Ca*. Thiosymbion oneisti A) and FLUY00000000 (*Ca*. Thiosymbion oneisti B). PCR-amplified *nifH* sequences can be found under accession nos. LT548937–LT548954 and partial 16S rRNA genes under LT548918–LT548936. The mass spectrometry proteomics data and the protein sequence database have been deposited at the ProteomeXchange Consortium via the PRIDE partner repository with the data set identifier PXD004536^80^.

## Supplementary information

Supplementary information Supplementary Discussion, Supplementary References, Supplementary Tables 1–4, Supplementary Figures 1–6, legends for Supplementary Datasets 1 and 2 (PDF 1688 kb)

Supplementary Dataset 1 Transcriptome mapping results. (XLSX 1708 kb)

Supplementary Dataset 2 Symbiont proteins identified in metaproteomes. (XLSX 1007 kb)
