# Supplementary material for: Chemosynthetic symbionts of marine invertebrate animals are capable of nitrogen fixation
Source: Nat Microbiol. 2016 Oct 24;2(1):16195. doi: 10.1038/nmicrobiol.2016.195 (PMC6872982; doi:10.1038/nmicrobiol.2016.195)
Supplement: Supplementary information — Supplementary Discussion, Supplementary References, Supplementary Tables 1–4, Supplementary Figures 1–6, legends for Supplementary Datasets 1 and 2 (PDF 1688 kb) [file 41564_2017_BFnmicrobiol2016195_MOESM201_ESM.pdf]

nature microbiology
SUPPLEMENTARY INFORMATION
ARTICLE NUMBER: 16195 | DOI: 10.1038/NMICROBIOL.2016.195

Chemosynthetic symbionts of marine invertebrate animals are capable of nitrogen fixation

Jillian M Petersen, Anna Kemper, Harald Gruber-Vodicka, Ulisse Cardini, Matthijs van der Geest, Manuel Kleiner, Silvia Bulgheresi, Marc Mußmann, Craig Herbold, Brandon K. B. Seah, Chakkiath Paul Antony, Dan Liu, Alexandra Belitz, Miriam Weber

Supplementary Discussion

Confirming that the nif genes belong to the symbiont genomes

In the lucinid symbiont genomes, the nitrogenase gene cluster was always found on a large contig of between 407 kb and 3.8 Mb in length. These contigs always contained many other genes identical to their homologs in the other symbiont draft genomes. The presence of nif gene clusters in the lucinid symbiont genomes is therefore unlikely to be due to random contamination in the lucinid symbionts.

Unlike the *Ca*. Thiodiazotropha endoloripes draft genomes, most of which assembled on fewer than 20 contigs, the draft genome of *Ca*. Thiosymbion oneisti was highly fragmented and could not be assembled on fewer than 2026 contigs. To confirm that the nif genes belong to the *Ca*. Thiosymbion oneisti genome, we did a connectivity analysis of the best assembly (*Ca*. Thiosymbion oneisti A) using the software Bandage[1]. This analysis showed clear connections between the ribosomal RNA operon of *Ca*. Thiosymbion oneisti and the core nif genes

© 2016 Macmillan Publishers Limited, part of Springer Nature. All rights reserved.

within eight graph nodes, confirming the physical linkage between these gene clusters (Supplementary Figure 1).

Nitrogenase regulation in the *Loripes lucinalis* symbiosis

Nitrogenase transcripts or proteins could not be detected in all lucinid individuals analysed (Datasets 1 and 2). This could be due to 1) the absence of these genes in the symbiont populations in some host individuals, or 2) down-regulation of the nitrogenase by the populations of symbionts in some individuals. A recent study of the sulphur-oxidizing symbionts of deep-sea mussels showed that different strains of bacteria colonize these mussels, and that some metabolic capabilities such as hydrogen oxidation are only encoded by some symbiont strains[2]. In the lucinid symbionts, a number of observations indicate that our results reflect regulation of gene expression and not strain-level differences in nitrogen-fixing ability. Firstly, in all draft genomes, the nitrogenase gene cluster was found on a large contig (between 407 kb and 3.8 Mb in length), and the metagenome coverage of the nif gene cluster was similar to the rest of the genome, indicating that it is likely present in all symbionts in a particular host individual (for an example see Supplementary Figure 6). Secondly, although the nitrogenase genes were not among the 30 most abundantly expressed in three of the five individuals sequenced for transcriptomics, a few transcripts did map to these genes (Supplementary Dataset 1). Finally, using specific primers, we were able to amplify and sequence a ≈ 290 bp fragment of the *nifH* gene from DNA extracted from the same samples used for the transcriptome analysis. These sequences were 99%-100% identical to the *nifH* gene from *Loripes lucinalis* symbiont A, which we used as a reference for transcriptome mapping. This

indicates that at least the *nifH* gene is present in the symbionts of the clam individuals where few or no transcripts could be detected. Nitrogen fixation therefore seems to be tightly regulated by the *Ca*. Thiodiazotropha endoloripes, similar to other known nitrogen fixers (reviewed by[3]).

Our expression studies are consistent with the hypothesis that nitrogen fixation genes are upregulated when the symbionts experience nitrogen limitation. Expression of genes encoding transporters for ammonium and urea could only be detected in those individuals where nitrogenases could also be detected, both in our transcriptomics and proteomics studies (Figure 4, Supplementary Figure 4, Supplementary Datasets 1 and 2). In *Corynebacterium glutamicum*, a model organism for studies of the regulation of nitrogen metabolism, ammonium and urea transporters are strongly upregulated under ammonium-limiting nitrogen-fixing conditions[4].

Carbon and nitrogen fixation at the single-cell level

Nitrogen fixation is metabolically costly, requiring eight electrons and up to 16 molecules of ATP per molecule of N_2 reduced to NH_3 and H_2[5]. Most nitrogen-fixing bacteria rely on phototrophic or heterotrophic metabolism to power nitrogen fixation. However, exceptions are known, such as acidophilic iron oxidizers of the *Leptospirillum* group III from acid mine drainage biofilms, which can fix nitrogen and inorganic carbon using reduced iron as the sole energy source, without the addition of organic carbon[6]. Metabolic reconstruction from our draft genomes showed that the bivalve and nematode symbionts encode complete TCA cycles and genes for importing organic compounds (Figure 2).

Moreover, based on our transcriptomes, the complete TCA cycle was expressed in all five individuals of *Loripes lucinalis* sequenced (Supplementary Dataset 1). It is currently unclear whether single symbiont cells are capable of fixing nitrogen and carbon simultaneously, using the energy gained from sulfide oxidation. Nitrogen-fixing symbiont cells may supplement their energy needs by taking up organic carbon. Possibly, the symbiont population may be partitioned into two sub-populations: one fixing carbon, and the other fixing nitrogen, as has been shown for the phototrophic cyanobacterium *Crocosphaera watsonii*[7]. This could also explain our transcriptome results, which indicated that aerobic and anaerobic metabolisms function concurrently in the gills. A sub-population experiencing aerobic conditions might preferentially fix carbon, and a sub-population experiencing micro-aerobic or anaerobic conditions might preferentially fix nitrogen. Future experiments tracking the incorporation of N_2, CO_2 and organic compounds at the single-cell level, for example with nanoSIMS, would help to answer these questions.

References

1. Wick, R. R., Schultz, M. B., Zobel, J. & Holt, K. E. Bandage: interactive visualization of *de novo* genome assemblies: Fig. 1. *Bioinformatics* **31,** 3350–3352 (2015).
2. Ikuta, T. *et al.* Heterogeneous composition of key metabolic gene clusters in a vent mussel symbiont population. *ISME J.* (2015).
3. Dixon, R. & Kahn, D. Genetic regulation of biological nitrogen fixation. *Nat. Rev. Microbiol.* **2,** 621–631 (2004).

4. Silberbach, M. *et al.* Adaptation of *Corynebacterium glutamicum* to ammonium limitation: a global analysis using transcriptome and proteome techniques. *Appl. Environ. Microbiol.* **71,** 2391–2402 (2005).
5. Karl, D. *et al. Dinitrogen fixation in the world's oceans*. (Springer Business and Science Media, 2002).
6. Tyson, G. W. *et al.* Genome-directed isolation of the key nitrogen fixer *Leptospirillum ferrodiazotrophum* sp. nov. from an acidophilic microbial community. *Appl. Environ. Microbiol.* **71,** 6319–6324 (2005).
7. Mohr, W., Vagner, T., Kuypers, M. M. M., Ackermann, M. & LaRoche, J. Resolution of conflicting signals at the single-cell level in the regulation of cyanobacterial photosynthesis and nitrogen fixation. *PLoS ONE* **8,** e66060 (2013).
8. Taylor, J. D. & Glover, E. A. Lucinidae (Bivalvia) - the most diverse group of chemosymbiotic molluscs. *Zool. J. Linn. Soc.* **148,** 421–438 (2006).

Supplementary Tables

Supplementary Table 1. Samples used in this study

Species	Location	Coordinates	Depth	Habitat	Sampling method	Purpose*	Date	Fixation
Laxus oneistus	Carrie Bow Cay, Belize	16°48′11’’ N, 88°04′55’’ W	1 m	Back-reef sand bar	Sieved from sediment	G	March 2014	RNAlater
Loripes lucinalis	Bay of Fetovaia, Elba, Italy	42°43‘49.0’’ N, 10°09‘20.5’’ E	7 m	Next to *Posidonia oceanica* seagrass bed	SCUBA diving, digging clams from sediment	G	20th Oct, 2010	RNAlater
						T	2nd Jun, 2015	RNAlater
						Pr	24th Oct, 2015	Frozen
						SI	21st and 24th Oct, 2015	Frozen
Loripes lucinalis	Thau Lagoon, France	43°26'53.95’’ N, 3°39'6.54" E	0.5 m	*Zostera noltii* seagrass beds	Sieved from sediment cores	P	12th Dec, 2015	96% Ethanol
Loripes lucinalis	Banc d’Arguin, Mauritania	19°53’0’’ N, 16°18’0’’ W	Intertidal	*Zostera noltii* seagrass beds	Sieved from sediment cores	SI	7th to 21st May, 2008	Frozen
		19°53’0’’ N, 16°18’0’’ W	Intertidal	*Zostera noltii* seagrass beds	Sieved from sediment	P	9th Dec, 2015	RNAlater
Codakia orbicularis	St George’s Cay, Bahamas	25°33'7.04" N, 76°45'56.85’’ W	Intertidal	*Thalassia testudinum* seagrass bed	Haphazard digging in seagrass sediment	P	24th Dec, 2015	RNAlater
Euanodontia ovum	Near boat jetty for Isle aux Aigrettes, Mauritius	20°25’26’’ S, 57°45’23” E	Intertidal	*Halodule* and *Halophila* seagrass beds	Sieved from sediment	P	2nd May, 2015	Absolute ethanol
Clathrolucina costada	Opposite Hyatt resort, SW Curaçao	12°03’57’’ N, 68°51’13’’ W	0.5 to 1 m	*Thalassia* and *Halodule* seagrass beds	Sieved from sediment	P	22nd May, 2015	Absolute ethanol

*Purpose: G = genomics, T = transcriptomics, Pr = proteomics, SI = stable isotopes, P = PCR amplification

Supplementary Table 2. Results of PCR screening in diverse lucinid species

Host subfamily§	Species	Sampling location	# of individuals	Sequences obtained*	
				**16S**	***nifH***
Lucininae	*Loripes lucinalis*	Mauritania	3	LT548935 - LT548936	LT548952 - LT548954
Lucininae	*Loripes lucinalis*	France	2	LT548933 - LT548934	LT548950 - LT548951
Lucininae	*Clathrolucina costata*	Curacao	1	LT548918	LT548937
Codakiinae	*Codakia orbicularis*	Bahamas	3	LT548919 - LT548921	LT548938 - LT548940
Pegophyseminae	*Euanodontia ovum*	Mauritius	1	LT548923	LT548941
Leucosphaerinae	*Epidulcina cf delphinae*	Madagascar	1	Only 16S rRNA could be amplified LT548922	
Codakiinae	*Lucinoma borealis*	Tjärnö, Sweden	1	Only 16S rRNA could be amplified LT548924	

§Subfamilies as defined by Taylor and Glover[8]

*Accession numbers

Supplementary Table 3. Stable isotope data used in this study

Location	Site	Latitude	Longitude	Date sampled	Species	ID	Shell length (mm)	Dry mass (mg)	δ^{13}C (‰)	δ^{15}N (‰)
Banc d'Arguin, Mauritania	1	19°53‘54.4’’ N	16°19‘49.4’’ W	18-May-08	*Pelecyora isocardia*	16950	6.5	0.216	-17.70	5.93
Banc d'Arguin, Mauritania	1	19°53‘54.4’’ N	16°19‘49.4’’ W	18-May-08	*Loripes lucinalis*	16951	7	0.224	-23.62	-1.07
Banc d'Arguin, Mauritania	2	19°53‘57.7’’ N	16°18‘36.6’’ W	10-May-08	*Pelecyora isocardia*	16411	14.8	0.277	-17.70	6.92
Banc d'Arguin, Mauritania	2	19°53‘57.7’’ N	16°18‘36.6’’ W	10-May-08	*Senilia senilis*	16414	57.8	0.239	-17.51	8.47
Banc d'Arguin, Mauritania	3	19°52‘36.7’’ N	16°15‘55.2’’ W	08-May-08	*Loripes lucinalis*	16226	10.9	0.230	-20.31	0.22
Banc d'Arguin, Mauritania	4	19°53‘33.2’’ N	16°18‘44.1’’ W	15-May-08	*Diplodonta circularis*	16688	14.2	0.216	-16.20	5.57
Banc d'Arguin, Mauritania	5	19°53‘26.3’’ N	16°19‘41.6’’ W	15-May-08	*Pelecyora isocardia*	16785	13.5	0.230	-17.80	6.36
Banc d'Arguin, Mauritania	5	19°53‘26.3’’ N	16°19‘41.6’’ W	15-May-08	*Senilia senilis*	16783	59.3	0.252	-19.58	7.84
Banc d'Arguin, Mauritania	6	19°53‘23.5’’ N	16°16‘46.3’’ W	08-May-08	*Pelecyora isocardia*	16258	19.3	0.246	-17.91	6.20
Banc d'Arguin, Mauritania	7	19°53‘38.9’’ N	16°16‘48.1’’ W	08-May-08	*Senilia senilis*	16251	40.6	0.251	-19.16	7.40
Banc d'Arguin, Mauritania	8	19°52‘47.8’’ N	16°16‘27.1’’ W	11-May-08	*Diplodonta circularis*	16448	9.6	0.192	-15.71	4.08
Banc d'Arguin, Mauritania	9	19°54‘32.9’’ N	16°19‘50’’ W	12-May-08	*Loripes lucinalis*	16496	6.7	0.322	-24.30	2.66
Banc d'Arguin, Mauritania	9	19°54‘32.9’’ N	16°19‘50’’ W	12-May-08	*Diplodonta circularis*	16498	12.2	0.297	-18.14	5.30
Banc d'Arguin, Mauritania	10	19°54‘52’’ N	16°18‘56’’ W	12-May-08	*Senilia senilis*	16480	16.1	0.251	-18.47	6.11
Banc d'Arguin, Mauritania	11	19°53‘36.5’’ N	16°16‘36.1’’ W	07-May-08	*Loripes lucinalis*	16221	9.9	0.213	-24.48	-3.67
Banc d'Arguin, Mauritania	12	19°52‘56.4’’ N	16°15‘26.1’’ W	08-May-08	*Pelecyora isocardia*	16262	10.6	0.226	-15.63	3.86
Banc d'Arguin, Mauritania	13	19°53‘54.4’’ N	16°18‘33.1’’ W	10-May-08	*Pelecyora isocardia*	16354	5.9	0.195	-15.14	6.90

Banc d'Arguin, Mauritania	13	19°53‘54.4’’ N	16°18‘33.1’’ W	10-May-08	*Senilia senilis*	16352	17.5	0.235	-16.01	7.93
Banc d'Arguin, Mauritania	14	19°53‘51.8’’ N	16°18‘52.9’’ W	10-May-08	*Loripes lucinalis*	16409	8.1	0.310	-25.43	-2.79
Banc d'Arguin, Mauritania	15	19°52‘52’’ N	16°16‘6.5’’ W	08-May-08	*Loripes lucinalis*	16233	6.5	0.262	-19.07	0.05
Banc d'Arguin, Mauritania	16	19°53‘23.8’’ N	16°19‘41.6’’ W	15-May-08	*Senilia senilis*	16778	56.1	0.208	-19.84	7.55
Banc d'Arguin, Mauritania	17	19°53‘12.7’’ N	16°15‘28’’ W	16-May-08	*Senilia senilis*	16881	16.3	0.247	-12.54	5.34
Banc d'Arguin, Mauritania	18	19°53‘23.9’’ N	16°15‘45.2’’ W	07-May-08	*Pelecyora isocardia*	16209	8.3	0.187	-15.17	5.27
Banc d'Arguin, Mauritania	18	19°53‘23.9’’ N	16°15‘45.2’’ W	07-May-08	*Senilia senilis*	16210	54.1	0.323	-15.21	7.83
Banc d'Arguin, Mauritania	19	19°52‘27.1’’ N	16°17‘25.4’’ W	15-May-08	*Loripes lucinalis*	16751	8.6	0.216	-24.42	-4.36
Banc d'Arguin, Mauritania	20	19°53‘44.9’’ N	16°19‘43.6’’ W	15-May-08	*Pelecyora isocardia*	16801	8	0.268	-15.62	5.50
Banc d'Arguin, Mauritania	21	19°54‘26.8’’ N	16°18‘58.3’’ W	14-May-08	*Pelecyora isocardia*	16621	6.3	0.282	-18.76	6.57
Banc d'Arguin, Mauritania	22	19°53‘40.7’’ N	16°16‘26.6’’ W	07-May-08	*Senilia senilis*	16214	54.4	0.221	-14.03	7.19
Banc d'Arguin, Mauritania	23	19°52‘52.9’’ N	16°15‘58.7’’ W	08-May-08	*Loripes lucinalis*	16259	10.4	0.205	-22.77	-1.23
Banc d'Arguin, Mauritania	24	19°50‘54.1’’ N	16°16‘10.7’’ W	21-May-08	*Diplodonta circularis*	18275	19.4	0.260	-13.61	4.82
Banc d'Arguin, Mauritania	25	19°52‘54.9’’ N	16°16‘58.9’’ W	11-May-08	*Senilia senilis*	16437	40.4	0.290	-18.30	7.34
Banc d'Arguin, Mauritania	26	19°53‘20.1’’ N	16°18‘53.5’’ W	13-May-08	*Loripes lucinalis*	16594	7.3	0.213	-24.42	3.34
Banc d'Arguin, Mauritania	27	19°53‘55.5’’ N	16°19‘41.9’’ W	18-May-08	*Pelecyora isocardia*	16966	17	0.189	-17.10	5.88
Banc d'Arguin, Mauritania	28	19°54‘36.1’’ N	16°19‘47.4’’ W	18-May-08	*Senilia senilis*	16945	30.8	0.332	-19.82	7.27
Banc d'Arguin, Mauritania	29	19°52‘48.5’’ N	16°16‘50.3’’ W	11-May-08	*Loripes lucinalis*	16424	6.9	0.303	-24.28	-3.64
Banc d'Arguin, Mauritania	30	19°53‘25.9’’ N	16°18‘44.9’’ W	15-May-08	*Senilia senilis*	16744	19.3	0.233	-17.39	7.35
Banc d'Arguin, Mauritania	31	19°53‘56.1’’ N	16°20‘11.2’’ W	13-May-08	*Diplodonta circularis*	16610	4.8	0.127	-18.55	5.72
Banc d'Arguin, Mauritania	32	19°52‘53.3’’ N	16°17‘2.3’’ W	21-May-08	*Senilia senilis*	18307	22.3	0.333	-18.66	7.10

Banc d'Arguin, Mauritania	33	19°53‘32.8’’ N	16°15‘50.5’’ W	07-May-08	*Loripes lucinalis*	16204	9.8	0.293	-23.75	-0.34
Banc d'Arguin, Mauritania	34	19°52‘31.9’’ N	16°17‘28’’ W	20-May-08	*Loripes lucinalis*	18266	5.6	0.248	-22.73	1.66
Banc d'Arguin, Mauritania	35	19°54‘56.9’’ N	16°18‘41.4’’ W	12-May-08	*Senilia senilis*	16526	55.7	0.244	-19.64	7.72
Banc d'Arguin, Mauritania	36	19°53‘19.4’’ N	16°16‘47.1’’ W	22-May-08	*Senilia senilis*	18321	60.9	0.335	-16.45	8.58
Banc d'Arguin, Mauritania	37	19°53‘29.8’’ N	16°18‘56.2’’ W	19-May-08	*Senilia senilis*	16988	36.1	0.250	-19.77	7.73
Banc d'Arguin, Mauritania	38	19°54‘28’’ N	16°19‘56.8’’ W	18-May-08	*Loripes lucinalis*	16956	9.3	0.286	-24.38	3.23
Banc d'Arguin, Mauritania	39	19°52‘43.6’’ N	16°16‘51.1’’ W	11-May-08	*Senilia senilis*	16429	48	0.249	-18.60	7.34
Elba, Italy	1	42°43‘49.0’’ N	10°09‘20.5’’ E	21-Oct-15	*Loripes lucinalis*	2015D1-1	14	0.624	-26.40	2.52
Elba, Italy	1	42°43‘49.0’’ N	10°09‘20.5’’ E	21-Oct-15	*Loripes lucinalis*	2015D1-2	16.5	0.617	-27.44	-4.76
Elba, Italy	1	42°43‘49.0’’ N	10°09‘20.5’’ E	21-Oct-15	*Loripes lucinalis*	2015D1-3	14	0.305	-26.05	1.90
Elba, Italy	1	42°43‘49.0’’ N	10°09‘20.5’’ E	21-Oct-15	*Loripes lucinalis*	2015D1-4	11.2	0.306	-26.62	-2.90
Elba, Italy	1	42°43‘49.0’’ N	10°09‘20.5’’ E	21-Oct-15	*Loripes lucinalis*	2015D1-5	10.8	0.608	-25.80	1.27
Elba, Italy	1	42°43‘49.0’’ N	10°09‘20.5’’ E	21-Oct-15	*Loripes lucinalis*	2015D1-6	11.8	0.664	-27.44	-4.11
Elba, Italy	1	42°43‘49.0’’ N	10°09‘20.5’’ E	21-Oct-15	*Loripes lucinalis*	2015D1-7	9.3	0.412	-25.75	1.69
Elba, Italy	1	42°43‘49.0’’ N	10°09‘20.5’’ E	21-Oct-15	*Loripes lucinalis*	2015D1-8	11	0.405	-19.79	2.24
Elba, Italy	1	42°43‘49.0’’ N	10°09‘20.5’’ E	21-Oct-15	*Loripes lucinalis*	2015D1-9	10	0.637	-27.13	-5.04
Elba, Italy	1	42°43‘49.0’’ N	10°09‘20.5’’ E	21-Oct-15	*Loripes lucinalis*	2015D1-10	8.1	0.631	-26.38	-0.53
Elba, Italy	3	42°43‘49.0’’ N	10°09‘20.5’’ E	24-Oct-15	*Loripes lucinalis*	2015D3-22	6	0.587	-25.93	-1.81
Elba, Italy	3	42°43‘49.0’’ N	10°09‘20.5’’ E	24-Oct-15	*Loripes lucinalis*	2015D3-23	5.5	0.883	-25.36	-1.22
Elba, Italy	3	42°43‘49.0’’ N	10°09‘20.5’’ E	24-Oct-15	*Loripes lucinalis*	2015D3-24	5.5	0.800	-26.01	-1.06
Elba, Italy	3	42°43‘49.0’’ N	10°09‘20.5’’ E	24-Oct-15	*Loripes lucinalis*	2015D3-25	5.6	0.805	-25.60	0.10
Elba, Italy	3	42°43‘49.0’’ N	10°09‘20.5’’ E	24-Oct-15	*Loripes lucinalis*	2015D3-26	9	0.819	-27.26	-4.07
Elba, Italy	3	42°43‘49.0’’ N	10°09‘20.5’’ E	24-Oct-15	*Loripes lucinalis*	2015D3-27	5.7	0.679	-24.91	-2.25
Elba, Italy	3	42°43‘49.0’’ N	10°09‘20.5’’ E	24-Oct-15	*Loripes lucinalis*	2015D3-28	6.5	0.815	-25.28	-1.66
Elba, Italy	3	42°43‘49.0’’ N	10°09‘20.5’’ E	24-Oct-15	*Loripes lucinalis*	2015D3-29	6.2	0.746	-25.97	-0.87

Elba, Italy	3	42°43‘49.0’’ N	10°09‘20.5’’ E	24-Oct-15	*Loripes lucinalis*	2015D3-30	7	0.838	-26.85	-1.00
Elba, Italy	3	42°43‘49.0’’ N	10°09‘20.5’’ E	24-Oct-15	*Loripes lucinalis*	2015D3-31	6.2	0.885	-26.71	-0.08

Supplementary Table 4. Parameter estimates of the linear mixed-effect model with either nitrogen **(A)** or carbon **(B)** stable isotope ratios (‰) as the dependent variable.

		Estimate	SE	*P*
**(A)** *Nitrogen stable isotope ($\delta^{15}N$)*				
Intercept		-0.47	0.73	0.53
Species[1]				
	L. lucinalis (Italy)	-0.61	1.03	0.56
	S. senilis	7.87	0.75	<0.001
	P. isocardia	6.21	0.77	<0.001
	D. circularis	5.52	0.79	<0.001
Random effects				
	$\sigma_{\text{sampling site}}$	0.72		
	σ_{residual}	0.20		
Variance function				
	$\sigma_{L.lucinalis}$	12.72		
	$\sigma_{L.\ lucinalis\ (italy)}$	11.58		
	$\sigma_{S.\ senilis}$	1.00		
	$\sigma_{P.\ isocardia}$	2.88		
	$\sigma_{D.\ Circularis}$	0.00		
**(B)** *Carbon stable isotope ($\delta^{13}C$)*				
Intercept		-23.32	0.52	<0.001
Species[1]				
	L. lucinalis (Italy)	-2.61	1.00	0.01
	S. senilis	5.59	0.69	<0.001
	P. isocardia	6.39	0.77	<0.001
	D. circularis	6.91	0.97	<0.001
Random effects				
	$\sigma_{\text{sampling site}}$	1.11		
	σ_{residual}	1.55		

[1]Reference category: *L. lucinalis* (Mauritania)

Supplementary Figures

Supplementary Figure 1. Connectivity analysis of the *Laxus oneistus* A metagenome

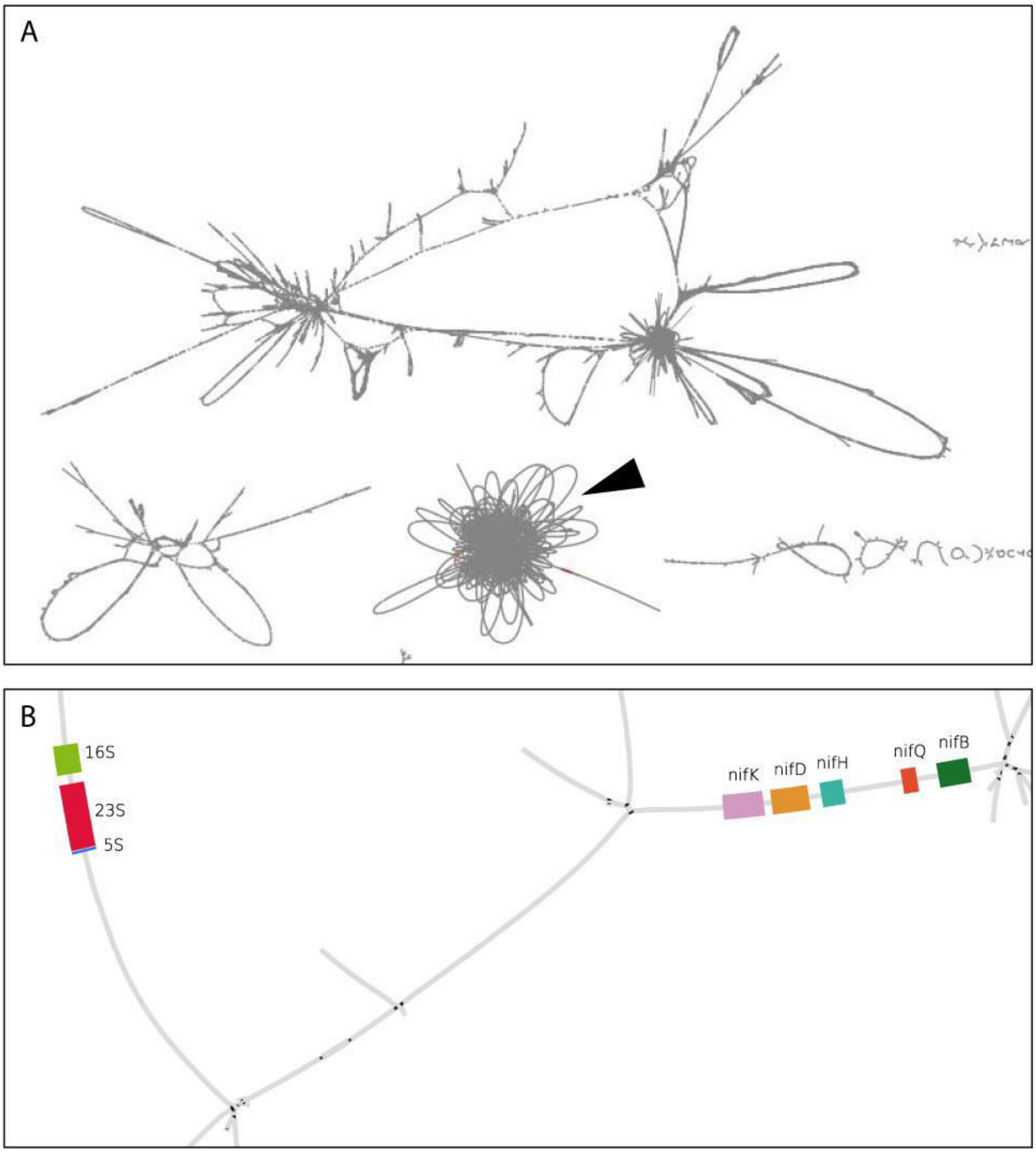

Supplementary Figure 1. Connectivity analysis of the *Laxus oneistus* A metagenome. A) Overview of the full assembly graph of the metagenomic assembly. The bin of connected *Ca*. Thiosymbion oneisti contigs is indicated with an arrowhead. B) Detailed view of the graph structure of the *Ca*. Thiosymbion oneisti bin that features the rRNA operon and the core nif operon within eight graph nodes from each other.

Supplementary Figure 2. Nif gene cluster in *Ca*. Thiodiazotropha endoloripes and comparison with *Azoarcus* sp. BH72

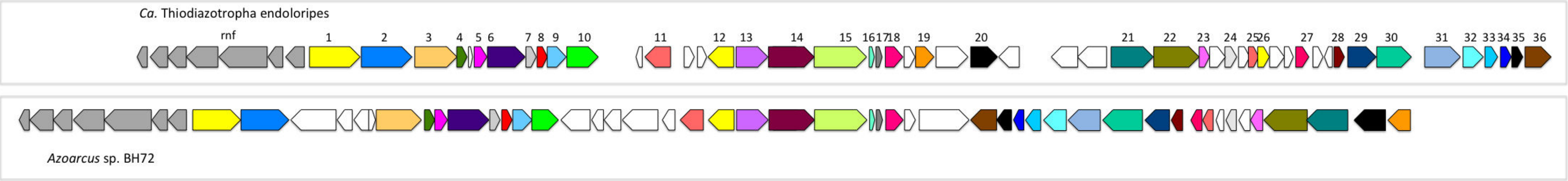

Supplementary Figure 2. Nif gene cluster in *Ca*. Thiodiazotropha endoloripes and comparison with *Azoarcus* sp. BH72

Schematic representation of the nif *and* rnf gene clusters in the *Ca*. Thiodiazotropha endoloripes D draft genome (NCBI Accession: LVJZ00000000), and in the model nitrogen-fixing bacterium *Azoarcus* sp. BH72 (NCBI Accession: NZ_LARU00000000). Homologs are shown in the same colour in each organism. Gene content and order are identical in all *Ca*. Thiodiazotropha endoloripes draft genomes (not shown). Genes encoding hypothetical proteins are shown in white. Genes with annotations are numbered as follows (RAST annotation):

1. Nitrogen fixation regulatory protein
2. Transcriptional regulator, NifA
3. FeMo-cofactor synthesis, NifB
4. 4Fe-4S ferredoxin, nitrogenase-associated
5. NifO
6. Probable flavoprotein
7. Ferredoxin
8. Ferredoxin
9. NifQ
10. ADP-ribosyl-(dinitrogen reductase) hydrolase
11. Conserved hypothetical protein

12. NAD(+)-dinitrogen reductase ADP-D-ribosyltransferase
13. Nitrogenase reductase asnd maturation protein NifH
14. Nitrogenase alpha chain, NifD
15. Nitrogenase beta chain NifK
16. NifT protein
17. 4Fe-4S ferredoxin, nitrogenase-associated
18. Dinitrogenase iron-molybdenum cofactor biosynthesis
19. LRV (FeS)4 cluster domain protein clustered with nitrogenase cofactor synthesis, leucine-rich repeat domain
20. Conserved hypothetical protein
21. Nitrogenase assembly protein NifE
22. Nitrogenase assembly protein NifN
23. Nitrogenase FeMo-cofactor carrier NifX
24. NifX-associated protein
25. Ferredoxin III, nif-specific
26. 2Fe-2S ferredoxin
27. Conserved hypothetical protein
28. Iron-binding protein in Nif operon
29. Nitrogenase Fe-S assembly scaffold protein NifU
30. Cysteine desulfurase NifS
31. Homocitrate synthase
32. Serine acetyltransferase
33. Conserved hypothetical protein
34. Nitrogenase stabilizing/protecting NifW
35. NifZ
36. NifM

Supplementary Figure 3. Phylogeny of NifD proteins

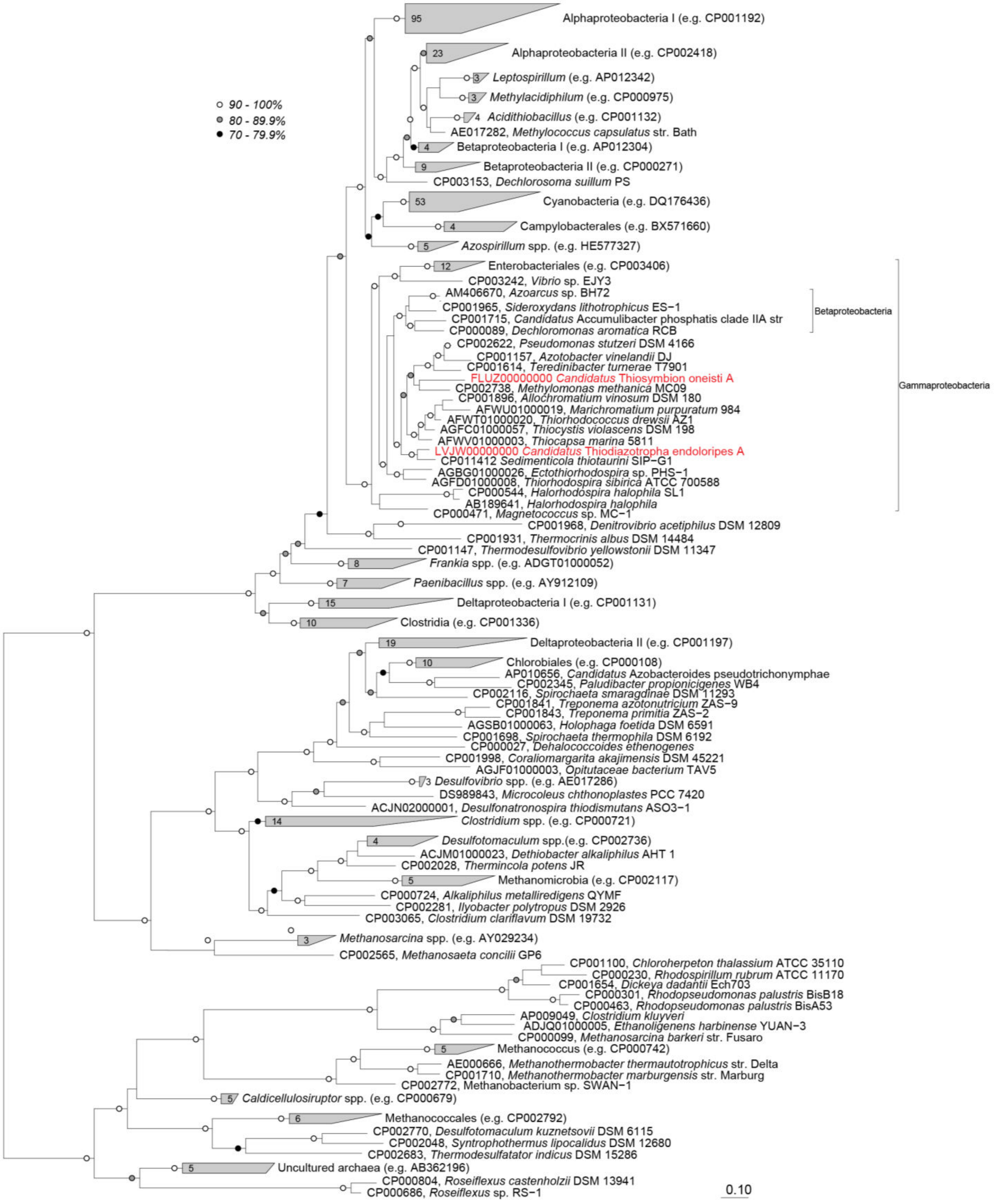

Supplementary Figure 3. Phylogeny of NifD proteins. Maximum likelihood phylogeny of full-length Group 1 NifH proteins. Percentages refer to bootstrap values. Sequences from this study are shown in bold red text.

Supplementary Figure 4. Top 30 most abundantly expressed genes in *Loripes lucinalis* (Elba) symbionts.

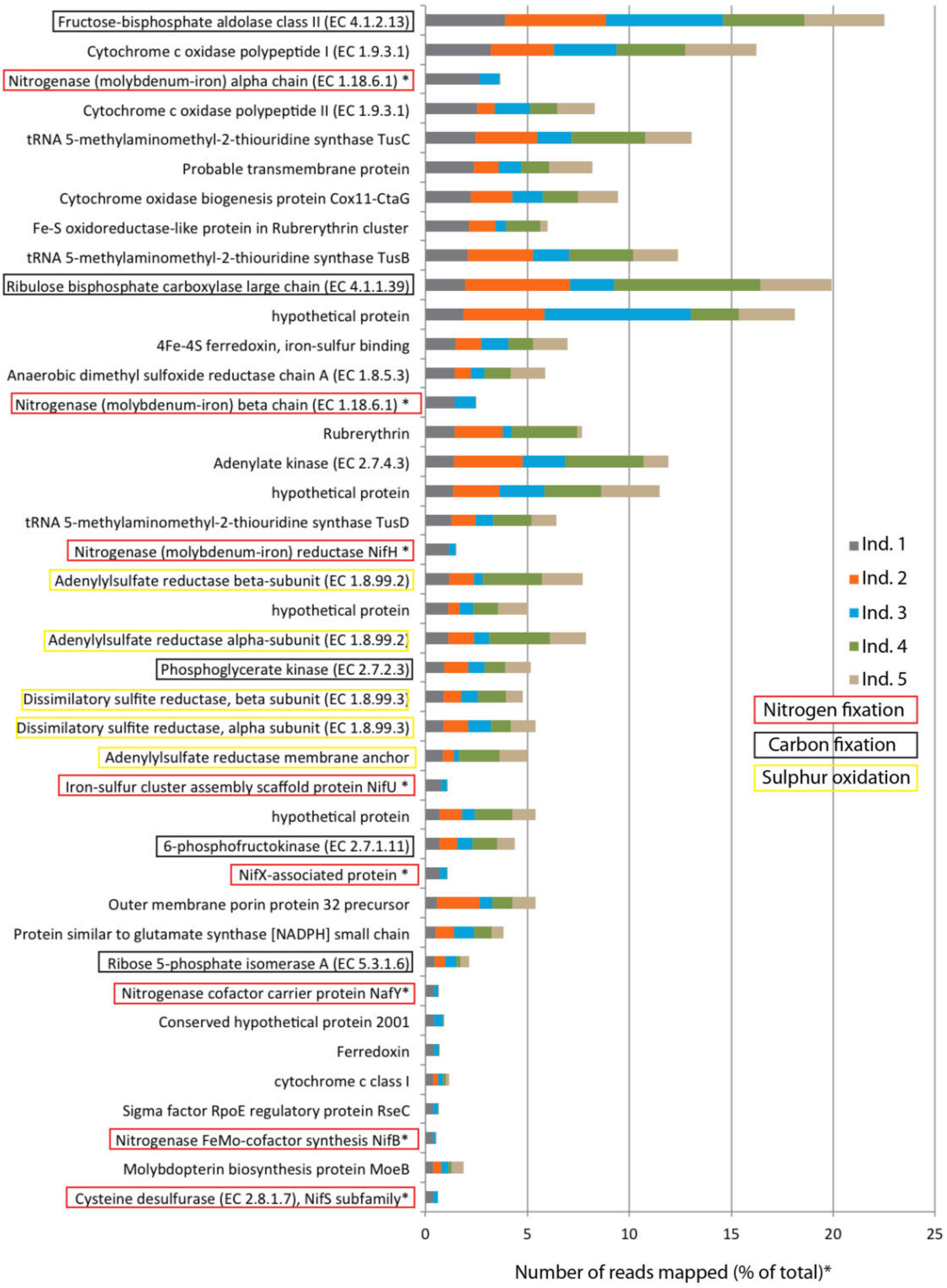

Supplementary Figure 4. Transcriptome identity plots. This figure shows the percentage of transcriptome reads (five transcriptomes from individuals 1 – 5)

that mapped to each of the five symbiont draft genomes (*Ca*. Thiodiazotropha endoloripes A – E), according to their nucleotide identity.

Supplementary Figure 5. Transcriptome identity plots

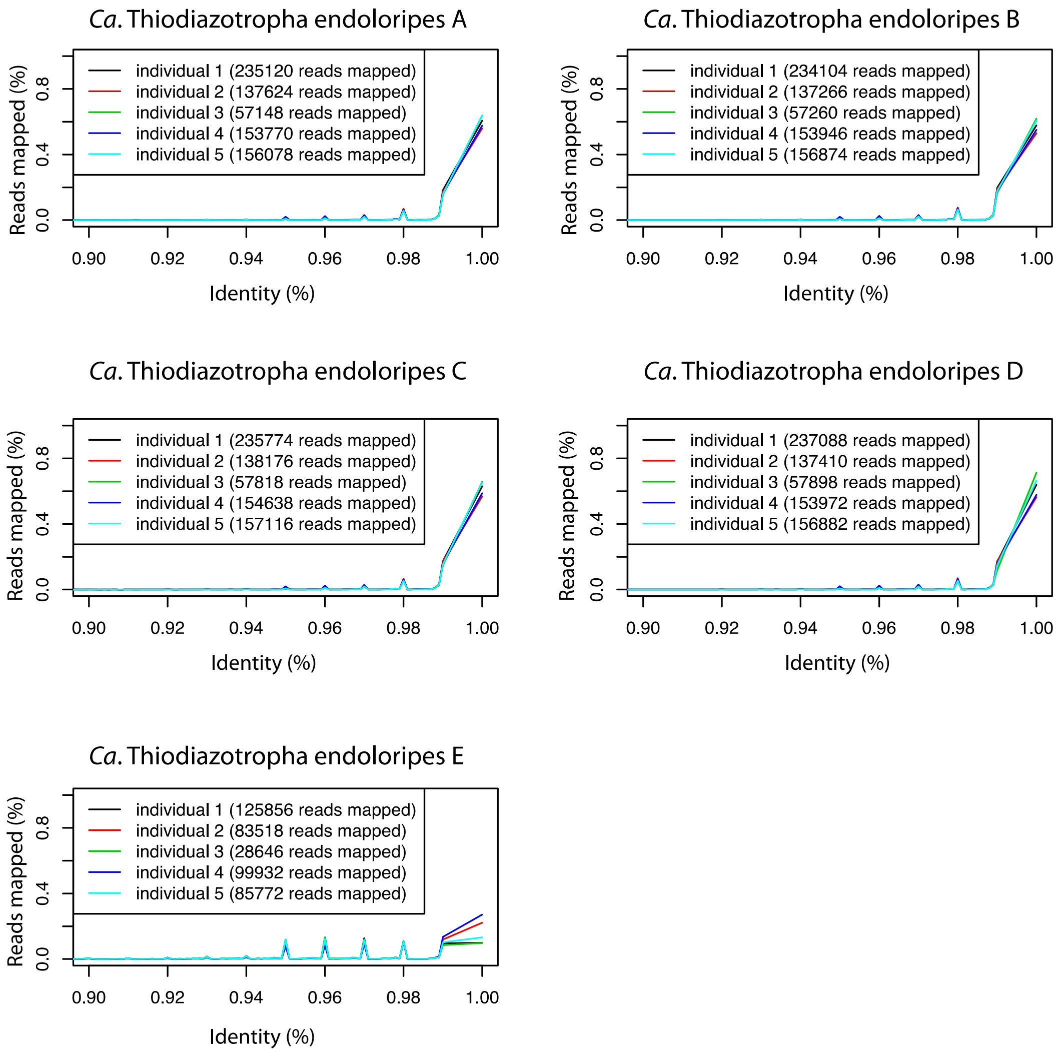

Supplementary Figure 5. Top 30 most abundantly expressed genes in *Loripes lucinalis* (Elba) symbionts. Bar chart of the 30 most abundantly expressed genes (*Number of reads mapped as a percentage of the total reads mapping to symbiont genes in one individual) in the symbiont transcriptome of *Loripes lucinalis* Individual 1 (grey bars), also showing the abundance of these 30 genes in the transcriptomes of individuals 2 to 5 (orange, blue, green and brown bars). Genes involved in nitrogen fixation are highlighted with an asterisk and indicated by a red box. Genes involved in carbon fixation are shown with a black box, those in involved in sulphur oxidation with a yellow box.

Supplementary Figure 6. Coverage plot of the contig containing the nitrogenase gene cluster in *Ca*. Thiodiazotropha endoloripes A.

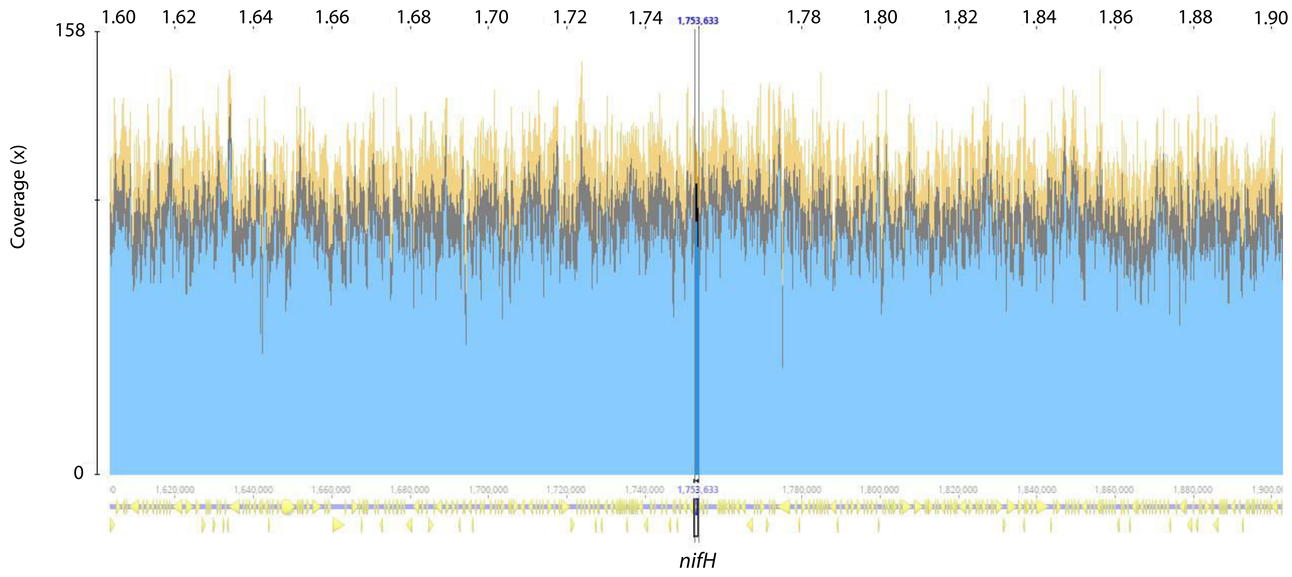

Supplementary Figure 6. Coverage plot of the contig containing the nitrogenase gene cluster in *Ca*. Thiodiazotropha endoloripes A. This plot shows the mapping of the metagenome reads from *Ca*. Thiodiazotropha endoloripes A to the portion of the contig containing the nif genes (*nifH* is marked with a vertical line). The contig is plotted on the x axis, with the contig position shown above (the entire contig is 2.4 Mb in length). The coverage (from 0 to 158 x) on the y axis. There is no clear drop in the coverage at the nif gene cluster compared to the surrounding regions, indicating that this gene cluster is present in all symbiont strains. Reads were mapped and visualized in Geneious (http://www.geneious.com/) .

Supplementary Dataset 1. Transcriptome mapping results – see separate Excel file 'Petersen_SupplementaryDataset1.xls'

This Excel file lists all predicted symbiont genes, and the number of transcripts that mapped to each gene in the five *Loripes lucinalis* individuals sequenced for transcriptomics. For easier comparison, RAST gene predictions are listed next to NCBI gene predictions.

Supplementary Dataset 2. Symbiont proteins identified in metaproteomes – see separate Excel file 'Petersen_SupplementaryDataset2.xls'

This Excel file shows all symbiont proteins identified in the six individuals of *Loripes lucinalis* whose gill proteomes were measured.
